# Transplantation of human stem cell-derived cone photoreceptors partially restores vision in aged *rd1* mice with advanced retinal degeneration

**DOI:** 10.1093/stmcls/sxag023

**Published:** 2026-05-02

**Authors:** Christopher A Procyk, Anna Melati, Menahil Tariq, Jingshu Liu, Matthew J Branch, Jamie D Delicata, Philippa Harding, Mahmoud Khazim, Majid Moshtagh Khorasani, Bryan Ladino, Emily P Lanning, Miriam Margari, Ifrax Mahamoud, Christi Mofidi, Krunal Narendra Kumar, Salome Van Heerden, Alexander J Smith, Emma L West, Robin R Ali, Rachael A Pearson

**Affiliations:** Ocular Cell and Gene Therapy Group, King’s College London Centre for Gene Therapy and Regenerative Medicine, Guy’s Hospital, London, SE1 9RT, United Kingdom; Ocular Cell and Gene Therapy Group, King’s College London Centre for Gene Therapy and Regenerative Medicine, Guy’s Hospital, London, SE1 9RT, United Kingdom; Ocular Cell and Gene Therapy Group, King’s College London Centre for Gene Therapy and Regenerative Medicine, Guy’s Hospital, London, SE1 9RT, United Kingdom; Ocular Cell and Gene Therapy Group, King’s College London Centre for Gene Therapy and Regenerative Medicine, Guy’s Hospital, London, SE1 9RT, United Kingdom; Ocular Cell and Gene Therapy Group, King’s College London Centre for Gene Therapy and Regenerative Medicine, Guy’s Hospital, London, SE1 9RT, United Kingdom; Ocular Cell and Gene Therapy Group, King’s College London Centre for Gene Therapy and Regenerative Medicine, Guy’s Hospital, London, SE1 9RT, United Kingdom; Ocular Cell and Gene Therapy Group, King’s College London Centre for Gene Therapy and Regenerative Medicine, Guy’s Hospital, London, SE1 9RT, United Kingdom; Ocular Cell and Gene Therapy Group, King’s College London Centre for Gene Therapy and Regenerative Medicine, Guy’s Hospital, London, SE1 9RT, United Kingdom; Ocular Cell and Gene Therapy Group, King’s College London Centre for Gene Therapy and Regenerative Medicine, Guy’s Hospital, London, SE1 9RT, United Kingdom; Ocular Cell and Gene Therapy Group, King’s College London Centre for Gene Therapy and Regenerative Medicine, Guy’s Hospital, London, SE1 9RT, United Kingdom; Ocular Cell and Gene Therapy Group, King’s College London Centre for Gene Therapy and Regenerative Medicine, Guy’s Hospital, London, SE1 9RT, United Kingdom; Ocular Cell and Gene Therapy Group, King’s College London Centre for Gene Therapy and Regenerative Medicine, Guy’s Hospital, London, SE1 9RT, United Kingdom; Ocular Cell and Gene Therapy Group, King’s College London Centre for Gene Therapy and Regenerative Medicine, Guy’s Hospital, London, SE1 9RT, United Kingdom; Ocular Cell and Gene Therapy Group, King’s College London Centre for Gene Therapy and Regenerative Medicine, Guy’s Hospital, London, SE1 9RT, United Kingdom; Ocular Cell and Gene Therapy Group, King’s College London Centre for Gene Therapy and Regenerative Medicine, Guy’s Hospital, London, SE1 9RT, United Kingdom; Ocular Cell and Gene Therapy Group, King’s College London Centre for Gene Therapy and Regenerative Medicine, Guy’s Hospital, London, SE1 9RT, United Kingdom; Ocular Cell and Gene Therapy Group, King’s College London Centre for Gene Therapy and Regenerative Medicine, Guy’s Hospital, London, SE1 9RT, United Kingdom; Ocular Cell and Gene Therapy Group, King’s College London Centre for Gene Therapy and Regenerative Medicine, Guy’s Hospital, London, SE1 9RT, United Kingdom; Ocular Cell and Gene Therapy Group, King’s College London Centre for Gene Therapy and Regenerative Medicine, Guy’s Hospital, London, SE1 9RT, United Kingdom; Ocular Cell and Gene Therapy Group, King’s College London Centre for Gene Therapy and Regenerative Medicine, Guy’s Hospital, London, SE1 9RT, United Kingdom

**Keywords:** retina, organoid, cone, transplantation, macular degeneration

## Abstract

Targeted photoreceptor replacement therapy is a promising, potentially disease-agnostic approach for reversing sight-loss associated with advanced retinal degenerations, including age-related macular degeneration. We have previously shown that transplantation of human stem cell-derived cone photoreceptors (hCones) into young adult (3-month-old) mouse models of advanced retinal degeneration can restore retinal function. However, substantial remodeling of the remaining inner retinal circuitry continues long after complete photoreceptor loss, raising the critical question of whether photoreceptor transplantation can effectively rescue function at very late-stage degeneration. *rd1* mice received transplants at 12-15 months of age and were examined ∼3 months later. Transplanted hCones survived in large numbers, while host inner retinal neurons exhibited significant plasticity, extending dendrites to transplanted hCones, and making synapse-like contacts. Host Müller glia undergo notable remodeling, apparently incorporating the donor cells within the retinal structure. Multielectrode array recordings showed robust rescue of light-evoked activity across the normal photopic range intensities and evidence of inner retinal processing, while some treated mice showed improvements in visually evoked optokinetic head tracking behavior. Together, these data indicate that effective rescue following photoreceptor replacement therapy is feasible long after complete photoreceptor loss and extensive inner retinal remodeling.

## Graphical abstract

**Graphical Abstract:**
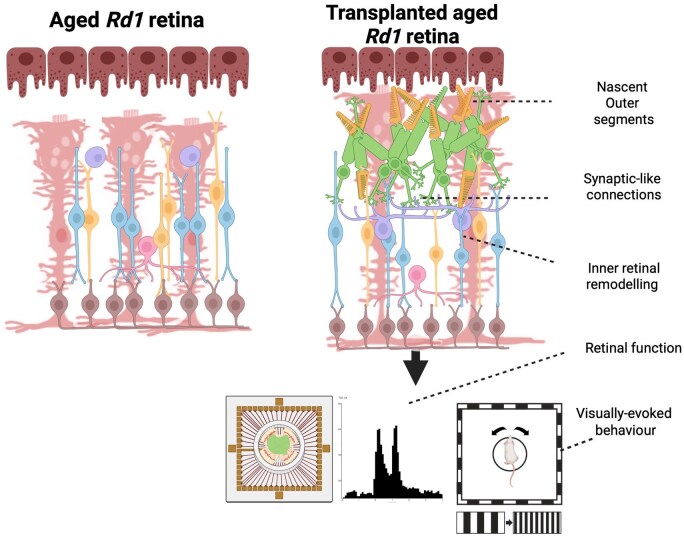

Significance statement Procyk et al. report the partial rescue of retinal and visual function in the aged (>1 year) *rd1* model of end-stage retinal disease by transplantation of stem cell-derived human cone photoreceptors, providing support for human cone transplantation as a therapy for very advanced retinal dystrophies.

## Introduction

Age-related macular degeneration (AMD) is the commonest cause of severe visual impairment in older adults in the developed world, with 67 million people in the EU currently affected.^1^ We live in an aging society and this figure is set to rise, with very significant personal and socio-economic consequences.^1^ In patients with AMD, impairment of high-acuity vision is primarily due to loss of foveal cone photoreceptor cells. Retinitis pigmentosa (RP) is a group of inherited retinal diseases characterized by progressive photoreceptor degeneration, vision loss, and can eventually result in total blindness in later life. The majority of genes associated with RP are rod-specific genes, but it is the secondary death of cones in the fovea that leads to loss of high-acuity vision that is ultimately most debilitating.^2^ Since the human retina, like much of the central nervous system, lacks the capacity to repair and regenerate, loss of the light-sensing photoreceptors is irreversible and there are few effective treatments. Cell replacement therapy is one of few therapeutic strategies with the potential to reverse, rather than slow, sight loss.^3^^,^^4^ Restoring functional neuronal connectivity by transplantation is ambitious in any disease context. However, the fovea is a very small cone-dense area (∼1.77 mm^2^) within the macula and replacement of even a small proportion of the 200 000 lost foveal cones may result in significant clinical benefit if there is effective functional connectivity.

We have previously reported that normal, healthy cone photoreceptors can be derived from human pluripotent stem cell (hPSC)-derived retinal organoids and that these may be isolated for transplantation purposes (herein termed “hCones”).^5^ When transplanted as a cell suspension into young adult (3-month-old) *rd1* and *aipl1^−/−^* models of advanced retinal degeneration, these hCones can repopulate an area of ∼2-2.5 mm^2^ in the degenerated photoreceptor layer.^6^^,^^7^ Transplanted hCones mature in vivo and stimulate the host inner retinal neurons to elaborate previously retracted dendritic processes, reexpress postsynaptic receptors and form new connections that are sufficiently robust to mediate restoration of retinal function in this region and drive some visually evoked behaviors.^6^^,^^7^ Others, taking a different, but related approach of transplanting pieces of partially intact neural tissue from retinal organoids, rather than a purified cell suspension, have also reported synaptic, retinal, and behavioral improvements.^8–12^ These collective findings are encouraging and continue to support the development of photoreceptor replacement therapy toward clinical application.^13^

However, an important and clinically relevant question remains; to what extent can functional rescue still be achieved when transplants are made into an aged retina, for example, in age-related degeneration or when transplants are made in retinas long after photoreceptor loss occurs, as may occur in end-stage RP. In many degenerations, the remaining inner retinal neurons continue to remodel, and even die, long after the photoreceptors have died^14–17^ and reactive gliosis, while variable between diseases,^18^^,^^19^ can eventually entomb the remaining cells potentially impeding donor-host incorporation.^20^^,^^21^ How this affects transplantation outcomes is not known. Here, we begin to address these questions by transplanting into the aged (>1-year-old) *rd1* mouse.^22^

## Experimental procedures

### Animals

Immunocompromised *Pde6b^rd1/rd1^/FoxN1^nu/nu^* (herein referred to as “*rd1*”^6^; Note that this *rd1* background does not carry confounding *Gpr179* bipolar cell mutation^23^) *C57BL/6J* wildtype and *Gnat1^−/−^*^24^ animals were maintained on standard 12 h light-dark cycle. Mice received food, water, and nesting material ad libitum. Male and female mice were used without discrimination and are represented in approximately equal numbers. All experiments have been conducted in accordance with the UK Animals (Scientific Procedure) Act of 1986 under PPL licence PP2007977 with approval by KCL’s local Animal Welfare and Ethical Review Body.

### hESC maintenance culture

The human H9 embryonic stem cell (hESC) line (WA09, female, ID/registry: WAe009-A [hPSCreg]; Lot RB66492, P30) was acquired directly from WiCell and used in accordance with the ISSCR Standards for Human Stem Cell Use in Research. In brief, a working cell bank (WCB) was cryopreserved at P33 in Knockout Serum Replacement (#10828010, ThermoFisher) with 10% CryoSure DMSO (#WAK-DMSO-10, WAK). These cells were fully characterized, including Karyometrix analysis (Stemnovate) with no major chromosomal changes detected (>60 bp). The undifferentiated state of the cell population was assessed by flow cytometry (Human Pluripotent SC Analysis kit, #560461 and #560589, BD Biosciences) with all pluripotent markers present in >70% of cells and differentiation markers present in <10% of cells. Each experimental run was initiated from freshly thawed vial of the WCB, with cells seeded for differentiation within 15 passages. ESCs were maintained under feeder-free conditions in Essential 8^TM^ media (#A1517001; ThermoFisher) on Geltrex (#A1413301; ThermoFisher) coated 6-well plates. They were fed daily and grown to between 60% and 80% confluence, before passaging with Versene (#15040066; ThermoFisher) and seeded at a density of 2 × 10^4^ cells cm^−2^. Routine mycoplasma (MycoAlert plus detection kit, #LT07-710, Lonza) and sterility testing (Tryptic Soy broth, #1463170010 and Fluid Thioglycollate media, #STBMFTM12, Merck) was performed monthly for all cultures.

### Retinal differentiation culture and photoreceptor transplantation

hESCs were maintained, differentiated into retinal organoids, transduced with *ShH10.2.1L/MOpsin.GFP* virus, and GFP+ hCones were isolated at 17-21 weeks of differentiation, as we have previously described,^5–7^ with minor modifications. GFP+ purity was ≥80% in all transplants (*n* = 3 sorts; flow cytometry postsort). Sorted cells were resuspended at a concentration of 5 × 10^5^/2 µL (viability at resuspension ≥97%) and injected into the subretinal space of the superior retina using a sterile 34-gauge hypodermic needle under direct visual control. All transplantations were performed by a single surgeon (R.A.P.). All physiological and histological assessments were performed 3-5 months posttransplantation, unless otherwise stated. Animals received transplantation in 1 eye only, the other eye remaining as an internal un-injected control. All animals underwent optokinetic reflex (OKR) testing, animal was excluded from analysis for persistent nonstimulus related head drift. Of the 10 *rd1*+hCone eyes, 6 were additionally processed for multielectrode array (MEA), of which 4 were recorded successfully and 2/6 were technical failures. The remainder were processed for IHC; 3 were lost due to fixation issues and an additional animal was transplanted and processed for IHC alone. It was, therefore, not possible to draw direct correlations between the different test modalities for any given animal.

### Retinal and visual function tests

#### Optomotor response

Optokinetic reflex was assessed using OptoDrum (Striatech). Mice were kept under ambient light conditions. Pupil dilation was not performed. The stimulus comprises a striped pattern rotating at 12 degree s^−1^ and 99.72% contrast.^7^ By changing spatial frequency, visual acuity thresholds were automatically determined in an unbiased manner. Maximum irradiance emitted by the screens as measured at the platform was 8.8 × 10^13^ photons cm^−2^ s^−1^. The reduced sensitivity of *Gnat1^−/−^* (cone-only) retinas, compared with wildtype, suggests a substantial rod contribution to the response at this irradiance, which is necessarily absent from the *rd1*+hCone and untreated *rd1* animals. Left and right eyes were tested separately. One *rd1* animal was discounted from OKR analysis due to a persistent head drift. Each eye was tested on 3-4 separate occasions within 1 week. Not all trials evoked responses in any given individual, most likely because only a small area of the retina is covered by the transplant and may not be stimulated if the mouse’s attention is directed away from the stimulus. As such, all individual trial outcomes from all animals assessed are shown in the relevant figure for full transparency. For statistical analysis, the mean acuity value per eye was determined and a Mann-Whitney *U*-test was used to test for significant differences between *rd1* untreated and *rd1*+hCone eyes. *n* = 9 *rd1+*hCones transplanted eyes, *n* = 9 *rd1* untreated eyes, *n* = 6 *Gnat1^−/−^* eyes, and *n* = 4 *C57BL/6* eyes were tested.

#### MEA: sample mounting

Eyes from *rd1+*hCone transplanted, untreated *rd1* and *gnat1^−/−^* (all >1 year old at time of recording) were enucleated and retinal dissection was performed under dim red lighting conditions in warm carboxygenated (95% O_2_, 5% CO_2_) AMES media supplemented with 1.9 g L^−1^ sodium bicarbonate (Sigma Aldrich). The retina was placed ganglion cell layer (GCL) down onto a perforated MEA (120pMEA100/30iR-ITO; Multi Channel Systems (MCS)). The MEA chamber was mounted into the head stage (MEA2100-120 head stage; MCS). Electrophysiological signals were digitized and recorded with a sampling frequency of 20 kHz. Tissue was perfused with carboxygenated AMES (PPS2; MCS) and 9-cis-retinaldehyde (100 μm; Sigma Aldrich) and maintained at 36 °C (TC02 controller; MCS). *n* numbers (eyes) refer to the number of successful MEA recordings made, as determined by background spiking activity being detected on the majority of electrodes (spike threshold detection at >3.5 SD and spike amplitude greater than 50 µV) prior to light stimulus presentation, indicative of a healthy retina and good electrical contact between retina and electrodes. We find poor contact with electrodes, and lack of spiking channels is often a result of difficulty in complete removal of the vitreous body in aged degenerate animals. *n* = 4 *rd1+*hCone transplanted, *n* = 3 untreated *rd1*, and *n* = 3 *gnat1^−/−^*.

#### MEA: presentation of visual stimuli

Full-field 1 s light steps were presented from darkness with a 10 s interstimulus interval and repeated 10 times using a Cyan LED (*λ*_max_ = 505 nm; M505L4; Thorlabs; Maximum irradiance at retinal surface = 1.12 × 10^15^ photons cm^−2^ s^−1^). For Pharmacology assessment of the origin of light responses, the glutamatergic blockers L-(+)-2-amino-4-phosphonobutyrate (L-AP4) (50 μM), 6,7-dinitroquinoxaline-2,3-dione (DNQX) (40 μM), and D-2-amino-5-phosphonovalerate (D-AP5) (40 μM) (all Sigma Aldrich) were added to the media for 20 min and 1 s light pulses repeated. Following this, the drugs were washed out for up to 1 h before repeating the light stimulation protocol.

#### MEA: data analysis

Neural waveforms were processed using Offline Sorter (v4.7.1; Plexon). In brief, principal component-based sorting was used to discriminate single units, identifiable as a distinct cluster of spikes in principal component space with a clear refractory period (>1 ms) in their interspike interval distribution. The identity of each animal’s experimental condition was known at the time of recording due to the need to place the GFP+ve transplanted cell mass over the array; however, whether each identifiable single unit is light responsive cannot be known at this stage of analysis. Single-unit data were subsequently sent to and stored in NeuroExplorer (v5.437; Nex Technologies) in preparation for further analysis.

#### MEA: identification of light responses

Spike sorted data in NeuroExplorer files were analyzed by custom written MATLAB codes reported previously.^6^^,^^7^ The peristimulus time histograms (PSTHs) were calculated with 100 ms bins. Thresholds were defined using both amplitude and duration of responses. For increases in firing rate, this was defined as the prestimulus baseline +3SD, while for decreases in firing rate the threshold was defined as the prestimulus baseline +1SD. Baseline is defined as the average firing rate in the 2 s preceding the stimulus over 10 repeated trials. The rules used to assign each neuronal response to a specified class are described in full in reference.^6^ Single units were additionally checked manually to verify the classification and reproducibility of the response; the latter being classed as a reproducible change in firing rate in 5 or more of the 10 stimulus repeats. This ensures only highly reproducible photoreceptor-driven light responses were categorized for further analysis, while removing any units which showed spontaneous 10 Hz oscillatory activity, a well-characterized hallmark of the *rd1* retina.^25^^,^^26^

#### MEA: latency and amplitude analysis

Amplitudes and latencies were calculated for individual light-responsive units. Amplitude is defined as the change in firing rate between light onset/offset and peak response. Latency is the time difference between stimulus onset/offset and peak response. Latencies were calculated from smoothed PSTHs with 10 ms bin to retain an appropriate time resolution, while amplitudes were calculated with a 100 ms bin.

#### MEA: pharmacology analysis

Single units were categorized to the 1 s light step, defined above. Peak firing rate for each light-responsive unit was then calculated under Pharmacology and Washout conditions and statistically analyzed for each unit using a 2-way ANOVA with Dunnett’s multiple comparison test.

### Measurement of cell mass area

Graft area was derived from fluorescent images taken using a dissecting microscope prior to mounting on the MEA array and only of those retinas used for MEA recording. These measurements are necessarily approximate, since the retina is minimally handled prior to recording to avoid disruption of donor-host contacts. Note the area covered by the transplant can often be larger or a different shape to the area of the MEA and so only a proportion of the cell mass is placed on the MEA; this means that it is not possible to directly correlate cell mass area with MEA outputs.

### Histology and immunohistochemistry

Some eyes were processed for immunohistochemistry; note, post-MEA retinas are of poor quality and unsuitable for histological processing and so direct comparisons between electrophysiology and histology in the same eyes could not be made. Eye cups were dissected, fixed in 4% PFA, cryopreserved in 20% sucrose, and embedded in OCT before cryo-sectioning at 12 µm thickness across 6 slides and stored at –20 °C. Immunohistochemistry was performed as previously described.^6^^,^^7^ A list of primary and secondary antibodies and staining conditions is provided in Table S1. Samples were imaged using a Zeiss LSM900 confocal microscope using 20 ×  and 40 ×  objectives. Images shown are maximum projection images (MIPs) of *xyz* stacks of representative fields of view, at resolution of 1024 × 1024 pixels and 1 µm intervals in the *z*-plane. Zeiss LSM image software, Imaris, ImageJ, and Adobe Photoshop were used for image processing. Wherever possible tissue sections within a given experiment were imaged with the same laser power, detector gain and offset settings.

### Statistical analysis

All values are presented as mean±SD unless otherwise stated; *n*, number of animals, retinas, or independent experiments performed, as appropriate; *n*, number of cells, images, or MEA single units examined, where appropriate. Statistical significance was assessed using Graphpad Prism software and denoted as *P<.05; **P<.01; ***P<.001. Appropriate statistical tests were applied including 2-way ANOVA with Bonferroni’s correction, and Wilcoxon Rank test, as specified in the text and/or figure legends.

## Results

### Inner retinal remodeling in the aged *rd1* mouse retina

**Figure 1.**
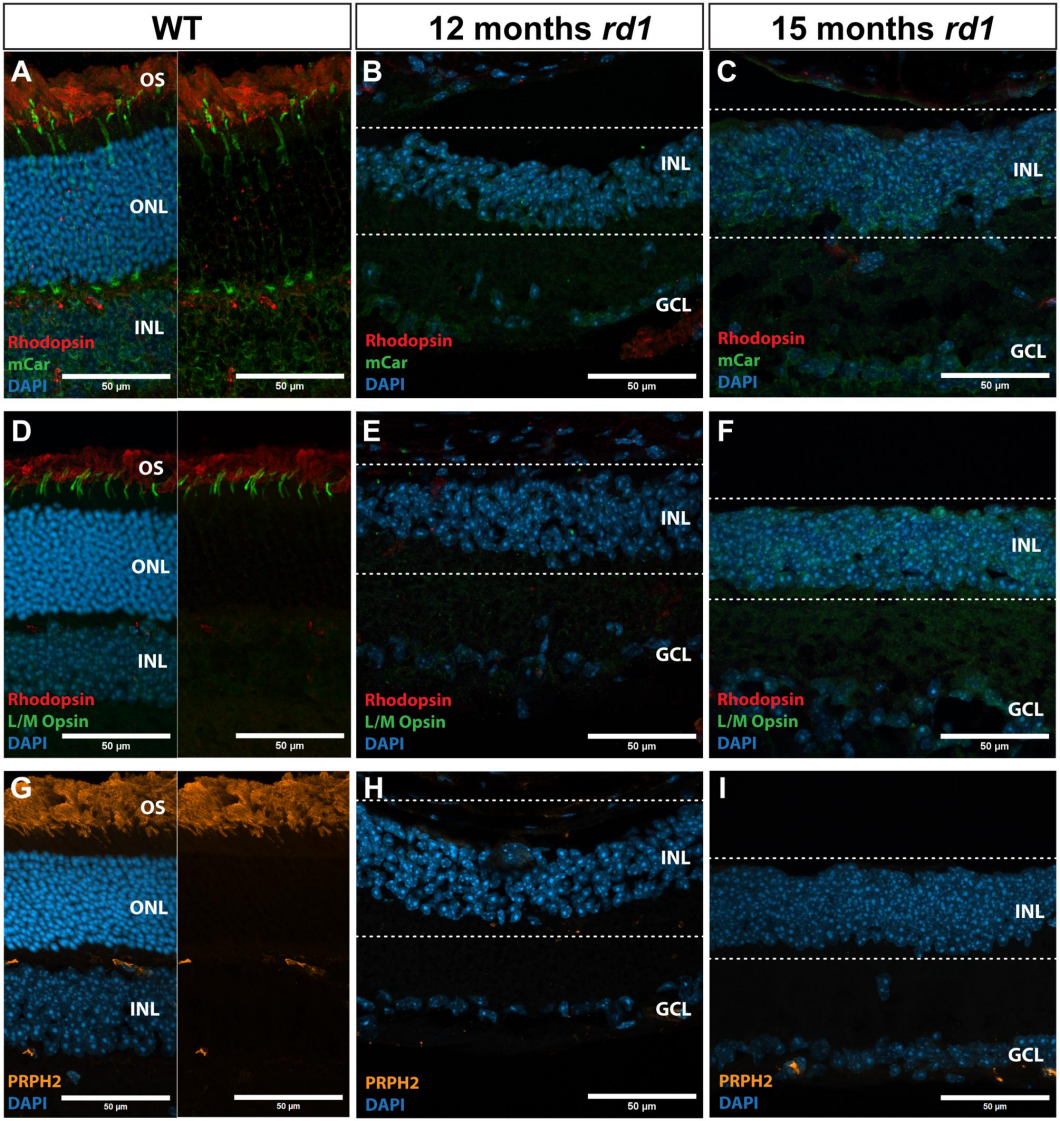
Outer nuclear changes in the aged *rd1* retina. Confocal MIPs of 12-month-old wildtype (WT) and *rd1* retina at 12 and 15 month of age. (A-F) Rods (Rhodopsin, red) and cones (mouse Cone Arrestin, green, panels A-C; L/M-Opsin, green, panels D-F) are present in large numbers in (A, D) 12-month-old wildtype mice and completely absent in (B, E) 12 and (C, F) 15 months of age in the *rd1* mouse; (G-H), Peripherin-2 (PRPH2, orange) clearly delineates the outer segments in (G) wildtype, but was absent in (H) 12- and (I) 15-month-old *rd1* mice. DAPI (blue) labels cell nuclei. Scale bars: 50 µm. Images are representative of *n*=3 eyes (wildtype), *n*=3 eyes (12 m untreated *rd1*), and *n*=3 eyes (15 m untreated *rd1*). Abbreviations: INL, inner nuclear layer; ONL, outer nuclear layer.

The *rd1* (*Pde6b^rd1/rd1^*) mouse is widely used as an animal model for autosomal recessive forms of RP.^27^ A naturally occurring mutation, also seen in patients,^28^ affecting production of the β subunit of phosphodiesterase 6 leads to accumulation of cGMP that leads to first rod and then cone photoreceptor degeneration. The *rd1* retina is very well characterized, exhibiting complete photoreceptor loss from the central retina by 3 months of age.^6^^,^^29^^,^^30^ Here, immunocompromised *rd1*/*FoxN1^nu^* (herein referred to simply as “*rd1*”) were assessed by immunohistological examination at 12 and 15 months of age (Figure 1). Accordingly, staining for Rhodopsin (Figure 1A-F), mouse-specific cone arrestin (mCar) (Figure 1A-C), L/M-opsin (Figure 1D-F), and outer segment protein, Peripherin-2 (Figure 1G-I), confirmed that all rods and cones had completely degenerated by 1 year, the age of the recipient mice at the time of transplantation (Figure 1A-I).

**Figure 2.**
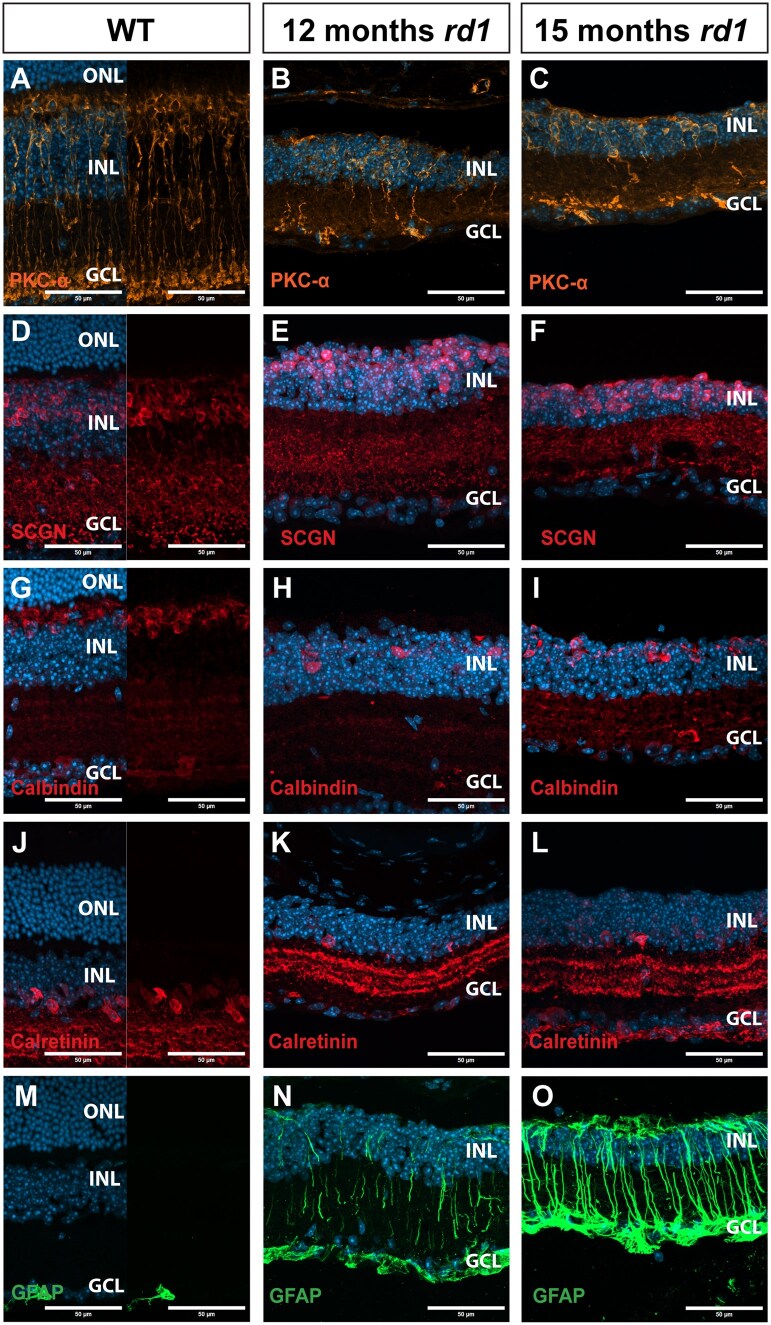
Inner nuclear changes in the aged rd1 retina. Confocal MIPs of 12-month-old wildtype and *rd1* retina at 12- and 15-months of age. (A-C) PKC-α positive ON bipolar cells, (D-F) Secretagogin (SCGN) positive ON and OFF cone bipolar cells, (G-I) Calbindin+ horizontal cells, (J-L) amacrine cells, stained for calretinin, and (M-O) GFAP+ (glial fibrillary acidic protein) reactive Müller glia and astrocytes. Both types of bipolar cells and horizontal cells show near complete retraction of dendritic arborizations in the *rd1.* Amacrine cells show limited changes, with some disorganization of IPL sublaminae. Müller glia show significant upregulation of GFAP in 12- and 15-month-old *rd1* retinas. DAPI (blue) labels cell nuclei. Scale bars: 50 µm. Images are representative of *n*=3 eyes (wildtype), *n*=3 eyes (12 m untreated *rd1*), and *n*=3 eyes (15 m untreated *rd1*). Abbreviations: INL, inner nuclear layer; IPL, inner plexiform layer; ONL, outer nuclear layer.

We next assessed remodeling occurring within the remaining inner retinal cell populations and compared this with age-matched wildtype mice (Figure 2). Immunostaining for PKCα labels both rod and cone ON bipolar cells, while immunostaining for secretagogin (SCGN) labels cone (ON and OFF) bipolar cells only.^31^ Both PKCα^+ve^ (Figure 2A-C) and SCGN^+ve^ (Figure 2D-F) bipolar cell dendrites appeared highly retracted in 1-year-old *rd1* mice, but the axonal projections and synaptic pedicles were largely maintained despite being without photoreceptor input for >11 months. Calbindin^+ve^ horizontal cell dendrites (Figure 2G-I) were also reduced. Notably, Calretinin^+ve^ amacrine cells appeared reduced in number, consistent with other reports, but the 3 sublaminae of the inner plexiform layer remained quite well defined even at 15 months (Figure 2J-L). Lastly, Müller glia exhibited extensive Gfap immunostaining throughout their extent (Figure 2M-O), consistent with our previous reports^18^^,^^29^; but we observed that this can vary between individual animals of the same age and strain (Figure S1). Together, these results show an ongoing process of dendritic retraction and some inner cell loss over time, after complete loss of photoreceptors.

### Transplanted human cones promote inner retinal remodeling and integrate with the aged *rd1* retina

Human cones were prelabeled in the organoid using *AAV-ShH10(Y445F)* containing *eGFP* under the control of the *2.1 L/MOPSIN* promoter (*L/MOPSIN.GFP*), which specifically labels human cones,^5^^,^^6^^,^^32^ and sorted for GFP fluorescence prior to transplantation. The purified hCones were transplanted into 12-15-month-old immunocompromised *rd1* mice; approximately 3 months posttransplantation, all animals were used for optomotor assessments. Subsequently, some eyes were processed for immunohistological examination, while others were processed for MEA recording.

**Figure 3.**
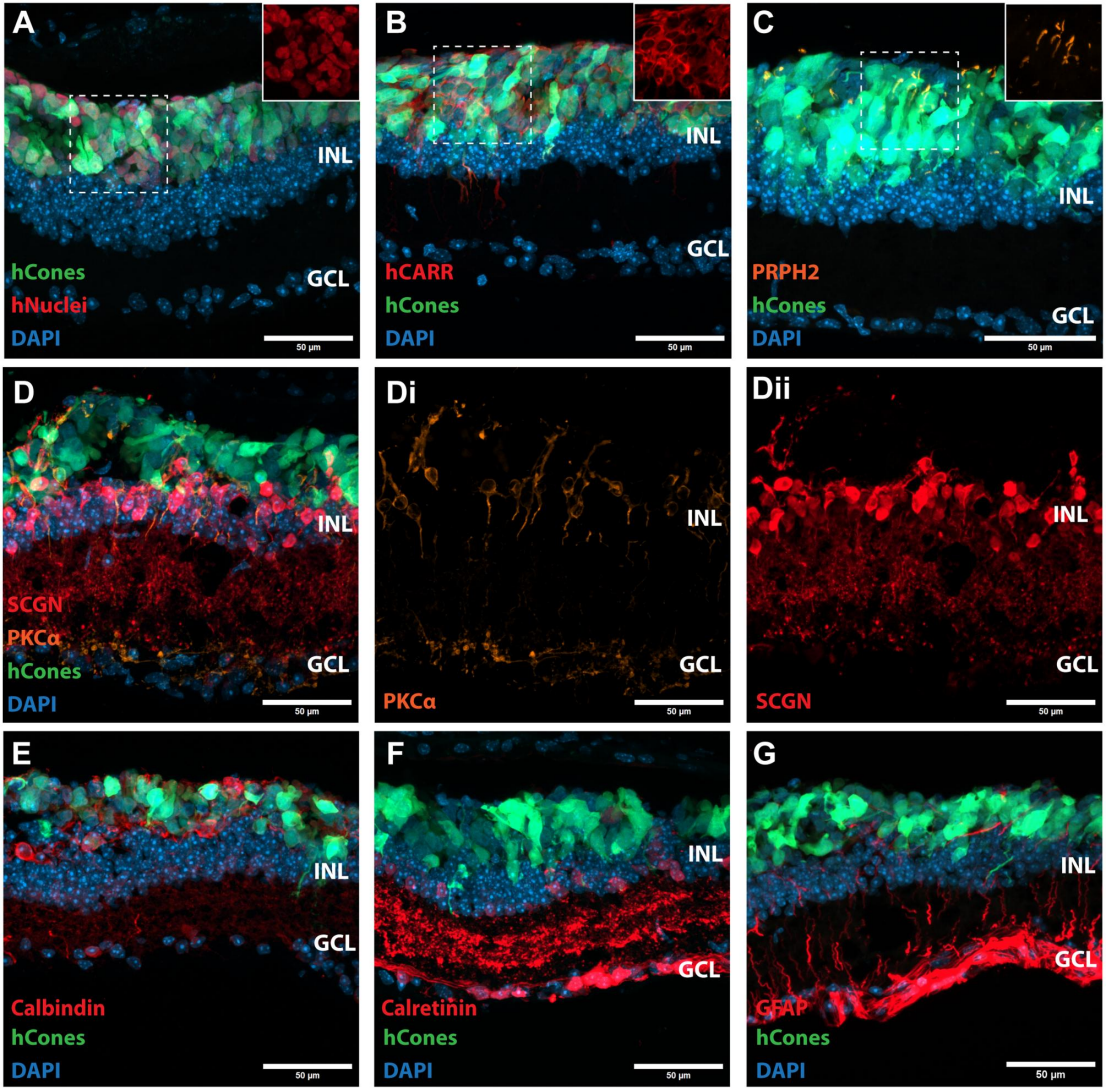
Human cones survive, mature, and promote reextension of inner retinal neuron dendrites in the aged *rd1* host retina. Confocal MIPs of *rd1* retina 3 months posttransplantation, at ∼15months of age. Large numbers of GFP+ hCones (green) could be seen in the subretinal space. These cells labeled for (Aii) human nuclear antigen (HNA; labeled hNuclei; gray) and (B) human cone arrestin (hCAR; red). All cells in cell mass were positive for HNA and hCAR staining, and all GFP+ cells labeled for both markers (A, B, inserts). (C) PRPH2+ (orange) bud-like structures, indicative of nascent segments, located toward the apical side of GFP+ hCone cell mass. See Figure S3 for further examples. (Di-ii) Both PKCα+ (orange) and SCGN+ (red) bipolar cells show reelaboration of apically directed dendritic processes, projecting toward and into the transplanted cell mass. (E) Calbindin+ (red) horizontal cells show widespread dendritic extension throughout much of the cell mass. (F) Calretinin+ (red) show limited changes, compared with controls. (G) GFAP+ processes extend up into and through the donor cell mass. See Figure S4 for further examples. DAPI (blue) labels cell nuclei. Scale bars: 50 µm. Images are representative of *n*=3 r*d1*+hCone transplanted eyes. Abbreviations: GCL, ganglion cell layer; INL, inner nuclear layer; ONL, outer nuclear layer.

Qualitative assessments showed that GFP^+ve^ hCones exhibited good survival in the aged *rd1* retina, similar to transplants made into younger *rd1* mice^6^ (Figure 3 and Figure S2). Shape and size of the area covered by the transplanted cell mass varied but covered an average area of 1.64 mm^2^±0.41 (*n* = 4 eye cups), based on those eyes processed as flatmounts for electrophysiology (see later). Staining of retinal sections for human nuclear antigen (HNA) (Figure 3A) and human-specific cone arrestin (hCAR) (Figure 3B) were present in almost all GFP^+ve^ cells confirming their human origin (we have previously excluded any contribution from material transfer^33^ in this model^6^). Numerous PRPH2^+ve^ outer segment-like structures were readily observed within the cell mass (Figure 3C and Figure S3). Notably, many of these were located on the apical side of the cell mass, the side nearest the host retinal pigment epithelium (RPE), indicating a degree of cellular polarization by the donor hCones.

Given the significant retraction of dendritic processes by host inner retinal neurons that continues after photoreceptor loss (Figure 2 and references,^14^^,^^15^^,^^18–20^^,^^30^), we examined whether they could still respond to the presence of healthy photoreceptors a year after the last endogenous photoreceptors had died. Remarkably, we saw extensive reelaboration of dendritic processes by PKCα^+ve^ ON bipolar cells (Figure 3Di, ii). SCGN^+ve^ cone bipolar cells also elaborated new dendrites up into the donor cell mass, although these appeared less numerous than PKCα^+ve^ dendrites (Figure 3Di, ii). Similar to our recent observations in the *Aipl1^−/−^* model of advanced disease,^7^ Calbindin^+ve^ horizontal cells also show an extensive elaboration of dendrites in the region underlying the hCone cell mass, and in some cases extending up into it (Figure 3E). No notable differences in Calretinin labeling were noted (Figure 3F), while many Müller glia from the host retina remodel, extending apical processes up through the hCone cell mass, seemingly incorporating it into the retinal structure (Figure 3G; see also Figure S4 for further examples, as the Müller glial response can be variable, even between litter mates^18^^,^^19^^,^^34^).

**Figure 4.**
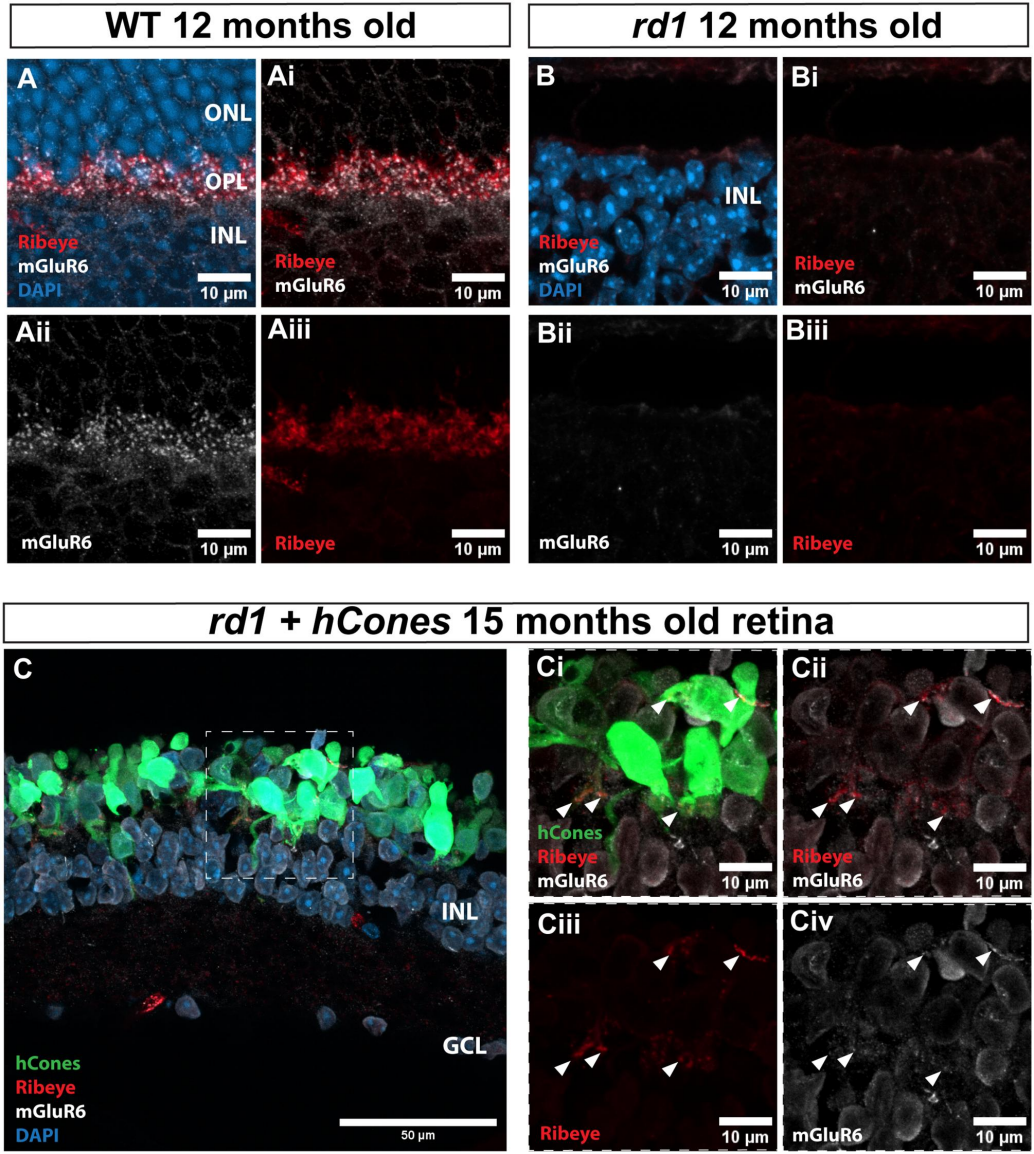
Human cones promote reexpression of postsynaptic mGluR6 and form nascent synaptic-like contact points with host BCs. Confocal MIPs of 12-month-old wildtype and *rd1* retina, and *rd1* retina 3 months posttransplantation, at 15 months of age. (A) immunostaining for presynaptic Ribeye (red), and postsynaptic mGluR6 (white) in 12-month-old wildtype mice. Ribeye is closely associated with mGluR6 (MIP of 3 *z*-sections). (B) In 12-month-old *rd1* retinas, there is little or no mGluR6 labeling and Ribeye is completely absent (MIP of 3 *z*-sections). (C) Three months posttransplantation of hCones (green) into 12-month-old *rd1* recipients, we observed widespread upregulation of mGluR6 signal. This included numerous puncta (gray) throughout the area of transplant as well as generalized signal in the cell bodies. Ribeye was also seen throughout the cell mass. (ROI) Ribeye+ (red) structures, present in transplanted GFP+ hCones, could be found in very close proximity to mGluR6+ puncta (white arrows). Scale bars: (A-B) 10 μm; (C) 50 μm and ROI, 10 μm. Images are representative of *n*=3 eyes (wildtype), *n*=3 eyes (untreated *rd1*), and *n*=3 eyes (*rd1*+hCone).

The photoreceptor presynaptic protein Ribeye, and the postsynaptic receptor, mGluR6, remain closely associated at the synaptic terminal in aged wildtype mice (Figure 4A). Ribeye is absent and mGluR6 is substantially downregulated in the *rd1* retina at 3 months,^6^ and, unsurprisingly, these are both virtually undetectable at 1 year (Figure 4B). However, by 3 months posttransplantation, many GFP^+ve^ processes contained Ribeye labeling and were in close proximity (<1 µm) to mGluR6^+ve^ puncta, which is indicative, albeit not conclusive, evidence of donor-host synapse formation (Figure 4C). Note that, in addition to the punctate labeling, we also observed a more diffuse cytoplasmic labeling with this mGluR6 antibody.

### Transplantation of human cones partially restores visually evoked optokinetic head tracking behaviors in some aged *rd1* retinae

**Figure 5.**
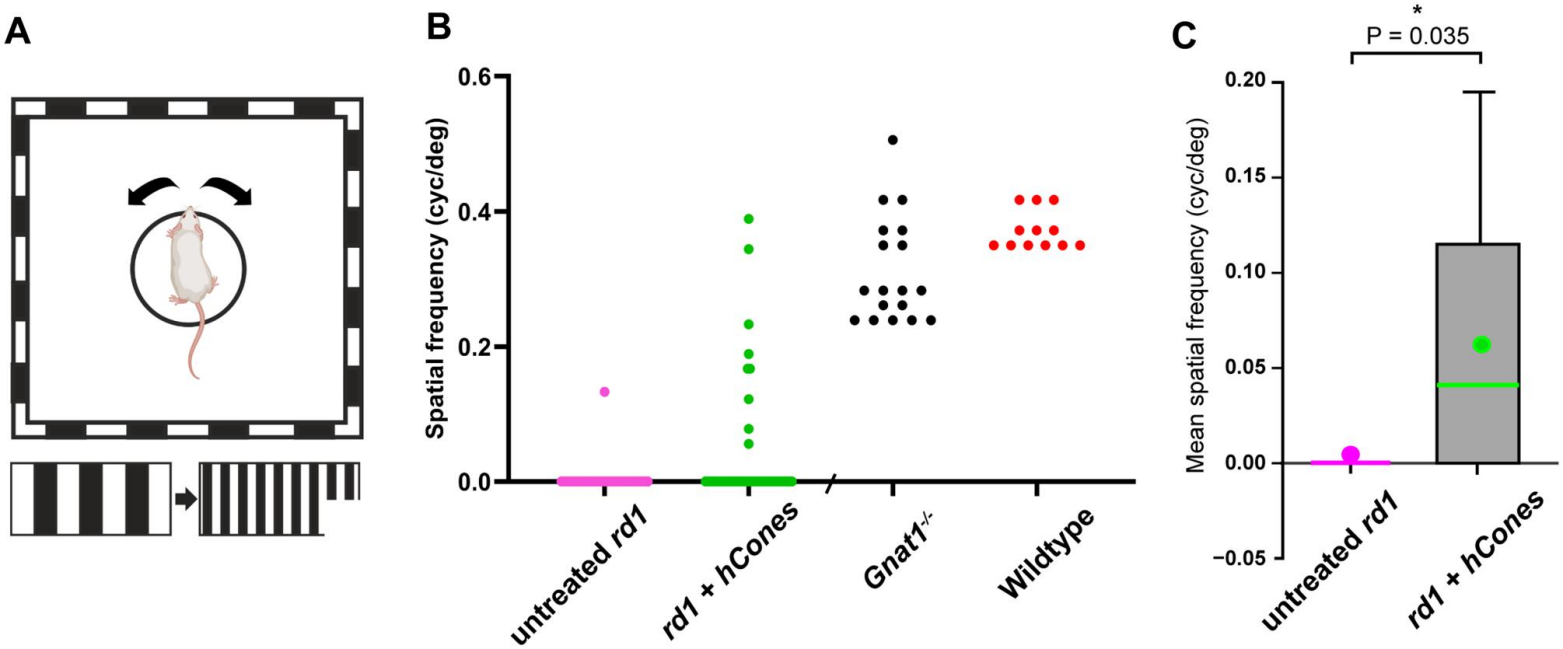
Transplantation of human cones restores optokinetic headtracking behaviors in the aged *rd1* retina. (A) Schematic of OptoDrum optokinetic headtracking setup. (B) Scatter plot of all individual acuity threshold measurements for 15-month-old hCone-transplanted (green) *rd1* eyes and contralateral untreated (magenta) eyes (*n*=9 eyes per group; 3-4 trials per animal, all trials shown; not all trials evoked a measurable response in any given animal). 12- to 15-month-old *gnat1^−/−^* (cone-only; black; *n*=6 eyes) and *C57Bl/6* wildtype (red; *n*=4) mice are shown for comparison. N.B. no head tracking behavior was seen in any untreated *rd1* mice, bar a single trial from 1 mouse. (C) Box-and-whisker plot of the mean acuity values per eye (see Table S1) for untreated *rd1* and *rd1*+hCone eyes. Magenta and green circles and lines denote mean and median values, respectively. A Mann–Whitney *U*-test was used to compare the mean acuity values of untreated *rd1* and *rd1*+hCone treated eyes; *P<.05.

We next sought to determine if and to what extent visual and retinal function could be rescued in the aged, end-stage retina. The optokinetic head tracking reflex (OKR) is a useful method for assessing visual function. The OptoDrum is a fully automated optomotor system, permitting assessment of the visual thresholds from both eyes independently (Figure 5A). In our previous study examining the transplantation of hCones in the *rd1* mouse,^6^ we had tried but had been unable to use this test due to software/camera constraints failing to correctly detect the immunocompromised nude mouse. These have since been addressed^7^ and we assessed OKR behaviors in aged *rd1* mice that had received hCone transplants in 1 eye (“*rd1*+hCone”) and tested ∼3 months later, the other eye remaining untreated. Each mouse was tested on 3-4 separate occasions within 1 week. One or more measurable visual acuity threshold value was detected in 5/9 *rd1*+hCone transplanted eyes. Optokinetic reflex thresholds were not detected in any untreated *rd1* eyes (*n* = 9), except for 1 recordable threshold from a single eye (Figure 5B), creating an otherwise clean baseline. Figure 5B shows the distribution of all recorded thresholds across all treated and untreated eyes (42 trials per group). Comparing the mean acuity values per eye (Figure 5C and Table S1), there was a modest but significant difference between the acuity thresholds measured in *rd1*+hCone transplanted eyes and untreated *rd1* eyes (*P* = .035; Mann–Witney *U*-test, *n* = 9). Responses from age-matched wildtype and *Gnat1^−/−^* mice (which provide a cone-only control as they lack rod α-transducin and therefore also lack rod function^24^) are shown for comparison.

### Transplantation of human cones restores light-evoked mERGs and spiking activity in the aged *rd1* retina

**Figure 6.**
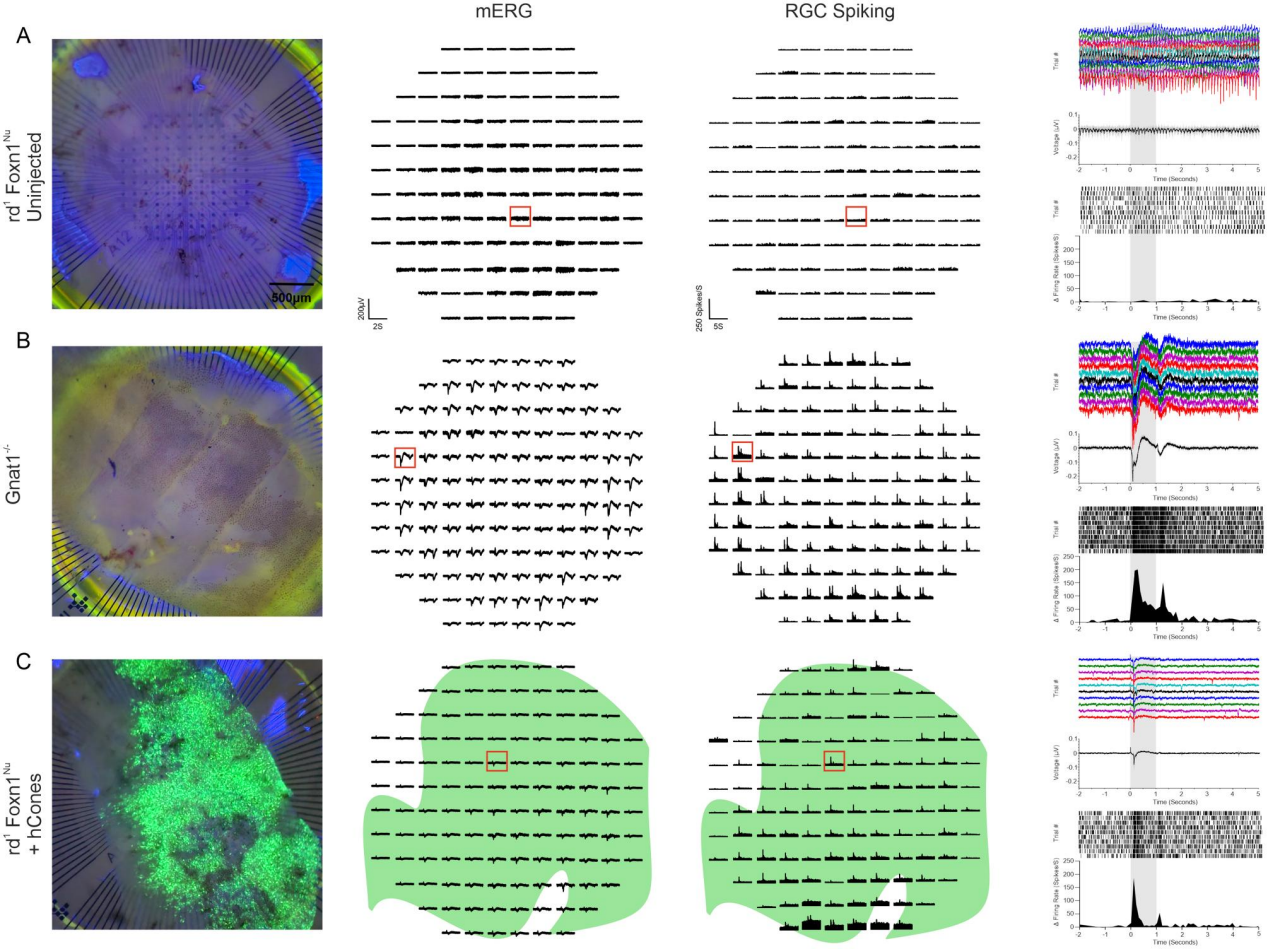
Transplanted human cones restore reproducible microERGs and multiunit spiking activity in the aged *rd1* retina. (A) (*left*) Representative untreated 12-month-old *rd1* retina on the MEA. There were no discernible light-evoked mERGs (*middle, left*), nor light-responsive spiking activity across the retina (*middle, right*). Red boxes in *middle panels* are magnified (*right)*, and show (*top*) mERG for 10 individual trials of a 1 s light pulse and (*bottom*) the mean (±SD), raster plot for multiunit (MU) spiking activity,and baseline subtracted PSTHs. No response to stimulus presentation was observed. (B) (*left*) Representative 12-month-old *Gnat1^−/−^* retina on the MEA. (*middle, left*) Most channels exhibit large amplitude light-evoked mERGs with a clearly defined *a* and *b* waves. (*middle, right*) Peristimulus time histogram (PSTH) of MU spiking activity demonstrated a wide variety of increases and/or decreases at light onset and/or offset across the majority of the array. Red boxes shown in middle panels are magnified (*right*) and show (*top*) reproducible mERG across 10 individual trials and (below) the mean (±SD), which are time locked to stimulus onset and offset, and (*bottom*) transient, large-amplitude changes in firing rate at light onset and/or offset in both the raster plot and baseline subtracted PSTH across the 10 individual trials. (C) (*left*) Representative hCone-transplanted retina. (*middle, left*) mERGs are seen on large proportion of channels and correlate with position of GFP^+^ cell mass (green overlay). (*middle, right*) PSTH of MU spiking activity demonstrated a wide variety of increases and/or decreases at light onset and/or offset on channels correlating with the position of the GFP^+^ cell mass (green overlay). Red boxes shown in middle panels are magnified (*right*) and show (*top*) discernible and reproducible mERG time locked to stimulus onset/offset and (*bottom*) the raster plot and baseline subtracted PSTH shows increases in firing rate at both light on/offset. Scale bars: (A, B, and C, *left*) 500 µm; (*middle, left*) 200 µV and 2 s; (*middle, right*) 400 spikes s^−1^ and 4 s. Gray bars denote 1 s light pulse. Examples shown are representative of *n*=3 eyes (*Gnat1^−/−^*), *n*=3 eyes (untreated *rd1*), and *n*=4 eyes (*rd1*+hCone).

Transplanted mice were subsequently used for either MEA recording or histology (see above). Micro electroretinogram (mERG) and multiunit (MU) spiking activity was recorded from the GCL in response to a 1 s uniform light step from darkness (Figure 6). As expected, untreated 1-year-old *rd1* retinas exhibited no discernible mERGs or changes in firing rate in response to the stimulus (Figure 6A; *n* = 3 retinas). Conversely, 1-year-old cone-only *Gnat1^−/−^* retinas (Figure 6B; *n* = 3 retinas) showed large amplitude mERGs in response to light steps, with clearly defined *a* and *b* waves. Multiunit firing also demonstrated an array of fast transient light responses at both light onset and/or offset when observing the PSTH. The same light stimulus was presented to *rd1+*hCone transplanted retinas (Figure 6C; *n* = 4 retinas), and in all cases yielded reproducible mERGs from the electrodes immediately under the GFP^+ve^ hCone donor cell mass (marked in green). This correlated strongly with fast transient increases in MU firing, which were time locked to stimulus onset and/or offset and again restricted to regions under the cell mass.

As we have observed previously,^6^^,^^7^ exposure to a cocktail of the synaptic blockers L-AP4, DNQX, and D-AP5, which block transmission of all known visual information at the photoreceptor/BC synapse,^35^^,^^36^ reversibly eradicated both mERG and all fast, transient responses at light onset and/or offset in *rd1*+hCone transplanted retinas (Figure S5). This supports the notion of glutamatergic-mediate transmission of light information between donor hCones and the host inner retina.

### Sensory characteristics of visual responses in aged *rd1* retina following human cone transplantation

**Figure 7.**
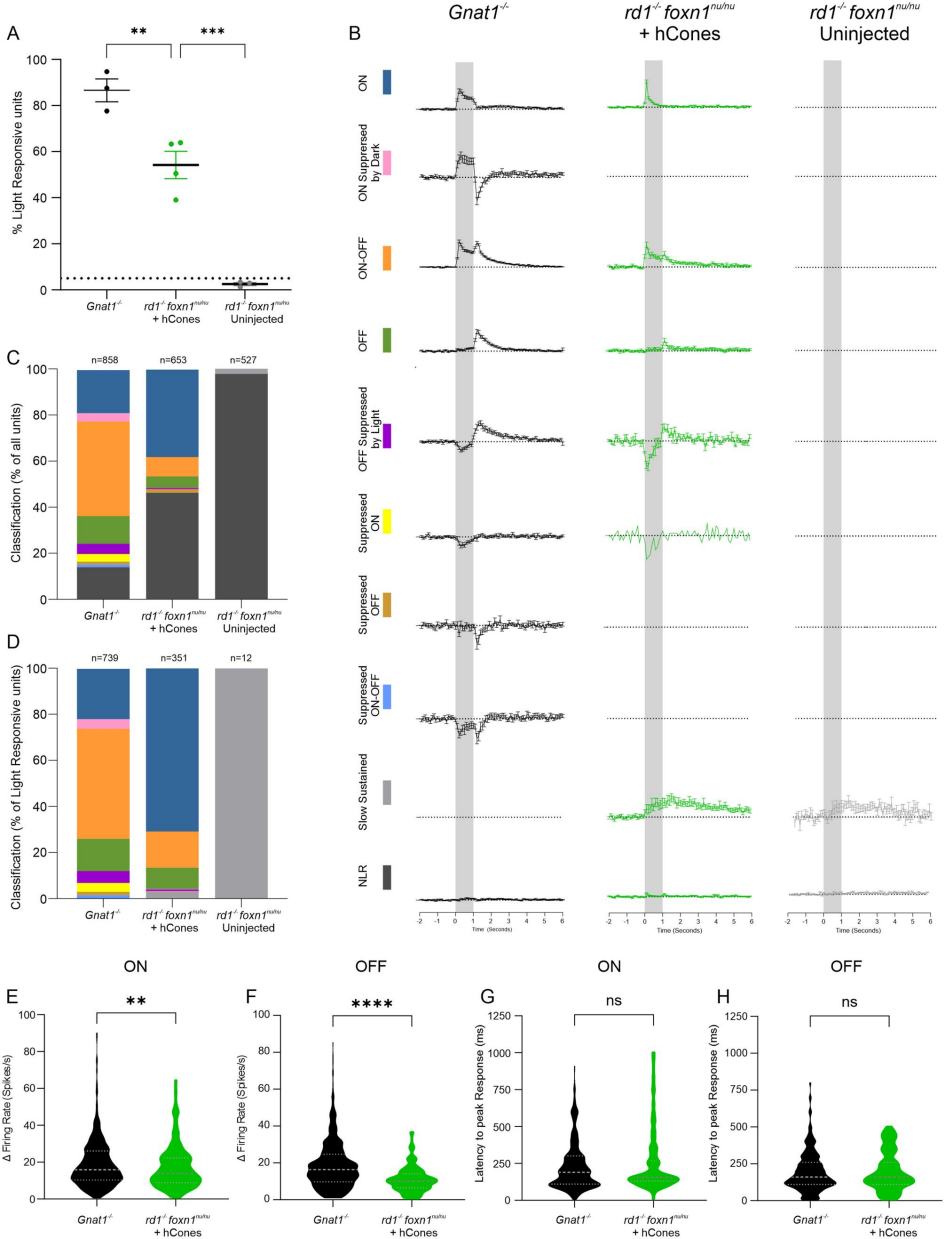
Transplanted human cones drive an array of fast, large-amplitude visual responses in the aged *rd1* retina. (A) Percentage of light-responsive units in aged *rd1*+hCone transplanted retinas (*n*=4 eyes) was lower than *Gnat1^−/−^* (*n*=3 eyes) but significantly higher than untreated age-matched *rd1* retinas (*n*=3 eyes) (1-way ANOVA). (B) Average PSTH of single units categorized into 10 quantitatively defined types based on their response to a 1 s light step in aged *rd1*+hCone and age-matched untreated *rd1* and *Gnat1^−/−^*. Time bin, 0.1 s; gray bars illustrate duration of light pulse. (C) Distribution of light-response types as percentage of all single units and (D) as percentage of all light-responsive units. Response types are color coded, as in panel (B). (E, F) Violin plots of response amplitude in aged *rd1*+hCone retinas (E) for ON-type responses (16.5±0.6 spikes s^−1^; *n*=304 units) and (F) OFF-type responses (10.7±0.7 spikes s^−1^; *n*=91 units) were significantly smaller than in *Gnat1^−/−^* retinas (19.2±0.6 spikes s^−1^, *n*=514 units and 18.8±0.6 spikes s^−1^, *n*=487 units; *P*<.0001 for both; unpaired *t*-test). (G and H) Violin plots of latency to peak response for ON and OFF components of light responses. Latency for ON-type responses in *rd1*+hCone retinas (235.5±10.8 ms) was not significantly different compared with *Gnat1^−/−^* retinas (234.1±7.5 ms; *P*=.915, unpaired *t*-test) or for OFF-type responses (196.2±13.7 ms vs 199.3±6.4 ms; *P*=.833, unpaired *t*-test). **P<.01, ***P<.001, ****P<.0001.

We have previously characterized the sensory characteristics of RGC responses in young *rd1* and *aipl1^−/−^* retinas transplanted with hCones using spike-sorting,^6^^,^^7^ and applied the same principles here. We found a significantly higher proportion of light-responsive units (54.2%±11.9) in *rd1* retinas receiving hCones (“*rd1*+hCones”), compared with untreated age-matched *rd1* retinas, (2.5%±1.2; *P*<.001; Figure 7A and C); this is similar to that which we reported previously for *rd1* mice receiving hCone transplants at 3 months of age (52.0%±9.0)^6^ but, as expected, lower than that seen in fully intact age-matched *gnat1^−/−^* retinas (86.6%±8.6; *P*<.01; Figure 7A and C).

We next classified the single light-responsive units based on their PSTH in response to a 1 s light step, as previously described.^6^^,^^7^ The mean (±SEM) PSTH of these 10 different response classifications are shown for *rd1+*hCones, untreated *rd1 and gnat1^−/−^* retinas (Figure 7B). Comparing the cohorts, and similar to what we have observed previously after transplanting hCones into 3-month-old *rd1* and *aipl1^−/−^* mice,^6^ a variety of transient and sustained, ON and OFF response types were observed in *gnat1^−/−^* mice and many of these were also seen in *rd1*+hCone mice (Figure 7B). Consistent with previous reports by us and others, the only responses to light observed in untreated *rd1* mice were slow, sustained responses, typical of the deafferented retina.^6^^,^^37^ In *rd1*+hCones mice, of the different responses seen, the most frequent type observed was ON responses (increased firing rate at light onset) (38.1% in 1-year-old *rd1+*hCones, compared with 21.8% in age-matched *gnat1^−/−^*) (Figure 7D). Only 8.42% of units could be characterized as ON-OFF in the 1-year-old *rd1+*hCones, compared with 41.4% in 1-year-old *gnat1^−/−^* retina, while OFF responses remained similar to *Gnat1^−/−^* (5.1% and 12.2%, respectively; Figure 7D). Suppressed-by-light ON and OFF responses were occasionally detected, but were comparatively rare, indicating more limited input of lateral pathways by the host retina, such as horizontal cells and amacrine, in modulating the glutamatergic-driven responses of the vertical pathway.

Examining response kinetics, the average response amplitude was smaller for both the ON and OFF responses in 1-year-old *rd1+*hCone transplanted retinas, compared with *gnat1^−/−^* cone-only controls (*P*<.01 and .001, respectively; unpaired *t*-test) (Figure 7E and F). Peak response latency for both ON and OFF responses was significantly different to that of *gnat1^−/−^* mice (Figure 7G and H; *P* = .915 and .833 for ON and OFF, respectively; unpaired *t*-test), showing that the transmission of visual information from transplanted hCones through the retina to the host RGCs is within the normal range.

## Discussion

Photoreceptor transplantation is gaining traction as a disease agnostic therapy with the potential to treat a variety of photoreceptor degenerations regardless of underlying cause. However, while we, and others, have shown the potential for rescuing visual function in a variety of animal models where no photoreceptors remain,^6^^,^^7^^,^^9^^,^^29^^,^^38^^,^^39^ the inner retina continues to remodel long after photoreceptor loss is complete.^14–17^^,^^40^^,^^41^ This raises important questions around how late in the degeneration process, and to what extent, photoreceptor transplantation in aged retina can bring about improvements in vision.

Degeneration-induced anatomical remodeling involves morphological changes of the remaining inner retinal neurons, including dendrite sprouting and retraction and in some cases cell kinesis, and has been described in AMD, inherited retinal dystrophies and retinal detachment.^14^^,^^15^^,^^20^^,^^42^ Concerns had been raised around the potential efficacy of cell replacement strategies to restore sight due to downregulation of mGluR6 receptors and a paradoxical upregulation of functional iGluRs on ON BCs in early degeneration,^40^^,^^43^^,^^44^ the continued remodeling of the inner retinal architecture in mid-degeneration,^14^^,^^15^ and the possible entombment of the remaining inner retina in late-stage degeneration (phase 3; as defined by Marc et al.).^14^^,^^18^^,^^20^^,^^21^ In this study, we examined structural and functional changes occurring in the recipient retina following transplantation of human cones in mice aged 1 year or older, finding them to be similar to those observed after transplantation into 3-month-old *rd1* and *aipl1^−/−^* mice,^6^^,^^7^ despite a further 9 or more months of inner retinal remodeling.

Our previous investigations into treatment of 3-month-old *rd1* and *aipl1^−/−^* mice^6^^,^^7^ were conducted after the aberrant, but transient, expression of iGluRs on rod and cone ON bipolar cells, which reportedly occurs between P25 and P100.^40^ In these prior studies, we showed reexpression of postsynaptic mGluR6 in host bipolar cells located immediately beneath the hCone transplant, but not distant to it, indicating that the presence of healthy photoreceptors stimulates this upregulation. Here, we show that mGluR6 is similarly reexpressed around the site of transplantation, often in close proximity to ribeye puncta in GFP+ hCones and that functional rescue, mediated by glutamatergic transmission, is still possible following transplantations at 12-15 months of age, well into late-stage (phase 3) degeneration. Together, these strongly support the notion that some form of synaptic-like contact and neurotransmission is achieved between hCones and the aged host inner retina. Although beyond the scope and resources of the current study, correlative electron microscopy will be of particular interest to further explore the nature of these putative synaptic contacts.

At least at the stages examined here, we saw no evidence of irreversible glial entombment; indeed, the contrary was observed, with many host Müller glia responding to the presence of the healthy hCones by extending apical processes up through the cell mass to incorporate it within, rather than separate it from, the host retinal structure. This is very encouraging for the treatment of aged retinas, even with ongoing gliosis. However, we know that Müller glial responses and scarring extent is variable, even between individuals suffering the same disease^18^^,^^19^^,^^34^ and we cannot rule out that it may still be problematic, particular if preventing retinal reattachment or if it becomes uncontrolled, for example, in massive retinal gliosis. Further work to examine the glial response to hCone transplants into a wider range of aged end-stage models is required to confirm this, along with a more in-depth ultrastructural investigation into whether host Müller glia can reform the OLM with donor photoreceptors.

The plasticity exhibited by the aged *rd1* retina in response to hCone transplantation is remarkable. However, we know that at least some inner retinal functionality is retained in advanced degeneration with a few remaining cones.^45^ Moreover, there is precedent for photoreceptors and inner retinal neurons retaining an intrinsic blueprint for connecting to one another, even in the absence of developmental cues: In an elegant study by Martemyanov and colleagues^46^ genetic manipulations were used to disconnect and reconnect rod photoreceptor/ON-BC synapses. In *Elfn1*-null mutants, synaptic contacts between rods and ON-BCs were abolished, compromising rod-mediated dim-light vision but leaving the wiring of cone pathways and cone-generated visual responses intact. By reintroducing *Elfn1* expression after the completion of synaptogenesis, they could induce connectivity and light-mediated function. Of note, BC dendrites appeared to be “plastic” in nature and could retarget to the rods that were expressing *Elfn1*. Moreover, the pathways involved in photoreceptor/BC synapse elaboration appear independent and *Elfn1* correctly localizes to the presynaptic terminus even in the absence of postsynaptic proteins like mGluR6. This is consistent with our observations in transplantation, where both host BCs and donor hCones form new dendritic and axonal processes, respectively, and Ribeye is often located to the axonal process, even if it is not always possible, with the tools available, to discern the postsynaptic partner.

A concern for cell replacement approaches is that we have little/no control over where donor and recipient neurons send out their axons and dendrites. Should these extend laterally, along the surface of the retina, this could have significant negative consequences for retinotopic mapping, which relies on the tight spatial organization between photoreceptors and the underlying bipolar cells and RGCs. Supporting the idea that at least some spatiotemporal processing occurs in the aged *rd1*+hCone transplanted mouse, OKR behavior and acuity measurements were similar to those recorded for 3-month-old hCone-transplanted *aipl1^−/−^* mice^7^ (OKR was not performed in the original 3-month *rd1* study^6^) and at least some threshold readings were in the same range as *gnat1^−/−^* cone-only controls. While undoubtedly partial and heterogeneous, such improvements are encouraging since transplantation rescues a relatively focal area of the retina and mice cannot be expected to specifically attend to complex visual stimuli arising in a small area of their (potential) visual field.

At the electrophysiological level, we again saw a similar proportion of units being reproducibly light responsive in aged mice as in the 3-month-old recipients we reported previously.^6^ RGC response amplitudes in transplanted retinas were unsurprisingly slightly smaller than those seen in *gnat1^−/−^* retinas, although this mostly reflected a reduction in the number of very large amplitudes, and the mean amplitudes were remarkably similar. Fewer large amplitude responses may result from the organization of donor photoreceptors, the number of photoreceptors connecting to bipolar cells, a reduction in the number of mGluR6 receptors expressed or some combination of the above. While we saw reasonably good orientation, with the majority of PRPH2+ outer segment-like structures located in the apical half of the graft, nearest the host RPEs, strategies to improve photoreceptor polarization, outer segment elongation and/or number of synaptic contacts could each improve response amplitude. Response latencies were similar to *gnat1^−/−^*.

The distribution of response types observed after spike-sorting was also broadly the same, although we observed fewer suppressed responses, possibly indicating changes in amacrine processing in these older, advanced disease retinas. We have shown that cell suspension transplants made into both the 3-month-old *rd1* and *aipl1^−/−^* retina exhibit a greater proportion of ON-type responses at the expense of ON-OFF types, compared with wildtype and *gnat1^−/−^* cone-only control animals.^6^^,^^7^ Very similar findings were recently reported after transplantation of partial tissue grafts derived from human retinal organoids.^8^^,^^11^ Here, in the aged *rd1* recipient, there appears to be a further increase in the proportion of ON-type responses, relative to other response types.

The differences we and others have seen between ON and OFF contributions in transplanted degenerated retinas, compared with wildtype, may reflect anatomical and/or functional changes within the remaining retinal circuitry, or the types of connections made between healthy hCones and that circuit. Degeneration-induced remodeling may mean ON and OFF information is processed differently in degenerating and transplanted retinas.^47^ We know that in some retinal degenerations, the photoreceptors and bipolar cells form new synaptic connections and alter their receptor expression patterns^17^; for example, in the slow degeneration *rd10* mouse retina, the OFF pathway becomes less excitable, apparently due to an increase in presynaptic inhibition.^48^ In addition, the known gap junction coupling of ON cone bipolar cells with the AII amacrine cell network in degeneration may alter the fidelity (polarity) of responses as they are transmitted through the retina.^49^ Of note, a recent study, again in the *rd10* model, Retinal Ganglion Cells (RGCs) with an OFF component to be more vulnerable than ON-type cells during progressive degeneration, with fast ON-type responses being most resilient to degeneration,^50^ consistent with our observations. Further investigations under light-adapted conditions with pharmacological interventions of these networks would more truly recapitulate the polarity of different RGC subtypes following transplantation.

In summary, we report the partial restoration of retinal and visual function following transplantation of hCones into the aged *rd1* mouse retina, indicating that treatment of late-stage degeneration may be possible. While retinal processing was evident, there were subtle but potentially important differences between mice treated at 3 months vs 1 year of age. Future studies using more complex and physiologically relevant visual stimuli will enable a better understanding of how this restored light sensitivity is processed both by the retina and the brain.

## Supplementary Material

sxag023_Supplementary_Data

## Data Availability

All unique/stable reagents generated in this study will be made available on request but may require payment and/or a completed Materials Transfer Agreement if there is potential for commercial application. *Rd1/Foxn1^nu^* mice will be provided directly, subject to availability. The datasets supporting the current study have not been deposited in a public repository because the authors are undertaking further analysis and any outputs arising from these will be published in due course but are available from the corresponding author on reasonable request. The codes used have been reported previously^6^ and will be shared by the lead contact upon request.
