# Supplementary material for: Transplantation of human stem cell-derived cone photoreceptors partially restores vision in aged rd1 mice with advanced retinal degeneration
Source: Stem Cells. 2026 May 2;44(7):sxag023. doi: 10.1093/stmcls/sxag023 (PMC13312030; doi:10.1093/stmcls/sxag023)
Supplement: sxag023_Supplementary_Data [file sxag023_supplementary_data.docx]

**SUPPLEMENTAL INFORMATION for:**

Transplantation of human stem cell-derived cone photoreceptors partially restores vision in aged *rd1* mice with advanced retinal degeneration

**Running title:** Human cones restore vision in aged *rd1* mouse

**Authors:** Christopher A. Procyk^1,2^, Anna Melati^1,2^, Menahil Tariq^1,2^, Jingshu Liu^1^, Matthew J. Branch^1,3^, Jamie D. Delicata^1,3^, Philippa Harding^1,3^, Mahmoud Khazim^1,3^, Majid Moshtagh Khorasani^1,3^, Bryan Ladino^1,3^, Emily P. Lanning^1,3^, Miriam Margari^1,3^, Ifrax Mahamoud^1,3^, Christi Mofidi^1,3^, Krunal Narendra Kumar^1,3^, Salome Van Heerden^1,3^, Alexander J. Smith^1^, Emma L. West^1^, Robin R. Ali^1,4^, Rachael A. Pearson^1,4^

1. Ocular Cell and Gene Therapy Group, King’s College London Centre for Gene Therapy and Regenerative Medicine, Guy’s Hospital, London, SE1 9RT, UK
2. Joint first authors
3. Equal contribution, listed alphabetically
4. Joint senior authors

***Author contributions***

(CP): Conception and design, Collection and/or assembly of data, Data analysis and interpretation, manuscript writing; (AM): Collection and/or assembly of data, Data analysis and interpretation, manuscript writing; (MT): Collection and/or assembly of data, Data analysis and interpretation, (JL): Collection and/or assembly of data, Data analysis and interpretation, (MB): Collection and/or assembly of data; (JD): Collection and/or assembly of data; (PH): Collection and/or assembly of data; (MK): Collection and/or assembly of data; (MK): Collection and/or assembly of data; (BL): Collection and/or assembly of data; (EL): collection and/or assembly of data; (M): Collection and/or assembly of data; (IM): Collection and/or assembly of data; (CM): Collection and/or assembly of data; (KNK): Collection and/or assembly of data; (SVH): Collection and/or assembly of data; (AJS): Conception and design, Data analysis and interpretation, manuscript editing; (EW) Conception and design, Collection and/or assembly of data, Data analysis and interpretation, manuscript editing; (RA): Conception and design, financial support, manuscript writing, final approval of manuscript; (RP): Conception and design, Data analysis and interpretation, financial support, manuscript writing, Final approval of manuscript

***Correspondence***

Co-corresponding authors:

Rachael Pearson, PhD, Ocular Cell and Gene Therapy Group, King’s College London Centre for Gene Therapy and Regenerative Medicine, Guy’s Hospital, London SE1 9RT, [rachael.pearson@kcl.ac.uk](mailto:rachael.pearson@kcl.ac.uk);

Robin Ali, PhD, Ocular Cell and Gene Therapy Group, King’s College London Centre for Gene Therapy and Regenerative Medicine, Guy’s Hospital, London SE1 9RT, [robin.ali@kcl.ac.uk](mailto:robin.ali@kcl.ac.uk)

***Funding and acknowledgements***

This work was supported by grants from the Medical Research Council UK (MR/T002735/2, MR/V038559/1, MR/R015651/1, MR/V030191/1, UKRI3826), and an unrestricted award from Guy’s and St Thomas’s Trust. Anna Melati is funded by the Wellcome Trust as part of the Advanced Therapies for Regenerative Medicine Wellcome Trust PhD Program (218461/Z/19/Z).

This work was made possible by the support, dedication and teamwork of the Ocular Cell and Gene Therapy group, and the Guy’s and St Thomas Clinical Trials Unit cell sorting facility. For the purpose of open access, the authors have applied a Creative Commons Attribution (CC BY) license to any Author Accepted Manuscript version arising. Schematics were generated using BioRender (https://biorender.com/).

***Key words***

Retina; organoid; cone; transplantation; macular degeneration

**Supplemental Information document contains:**

Supplemental Figures S1 – S5 and figure legends

Supplemental Tables S1, S2 and legends

**SUPPLEMENTAL FIGURES**


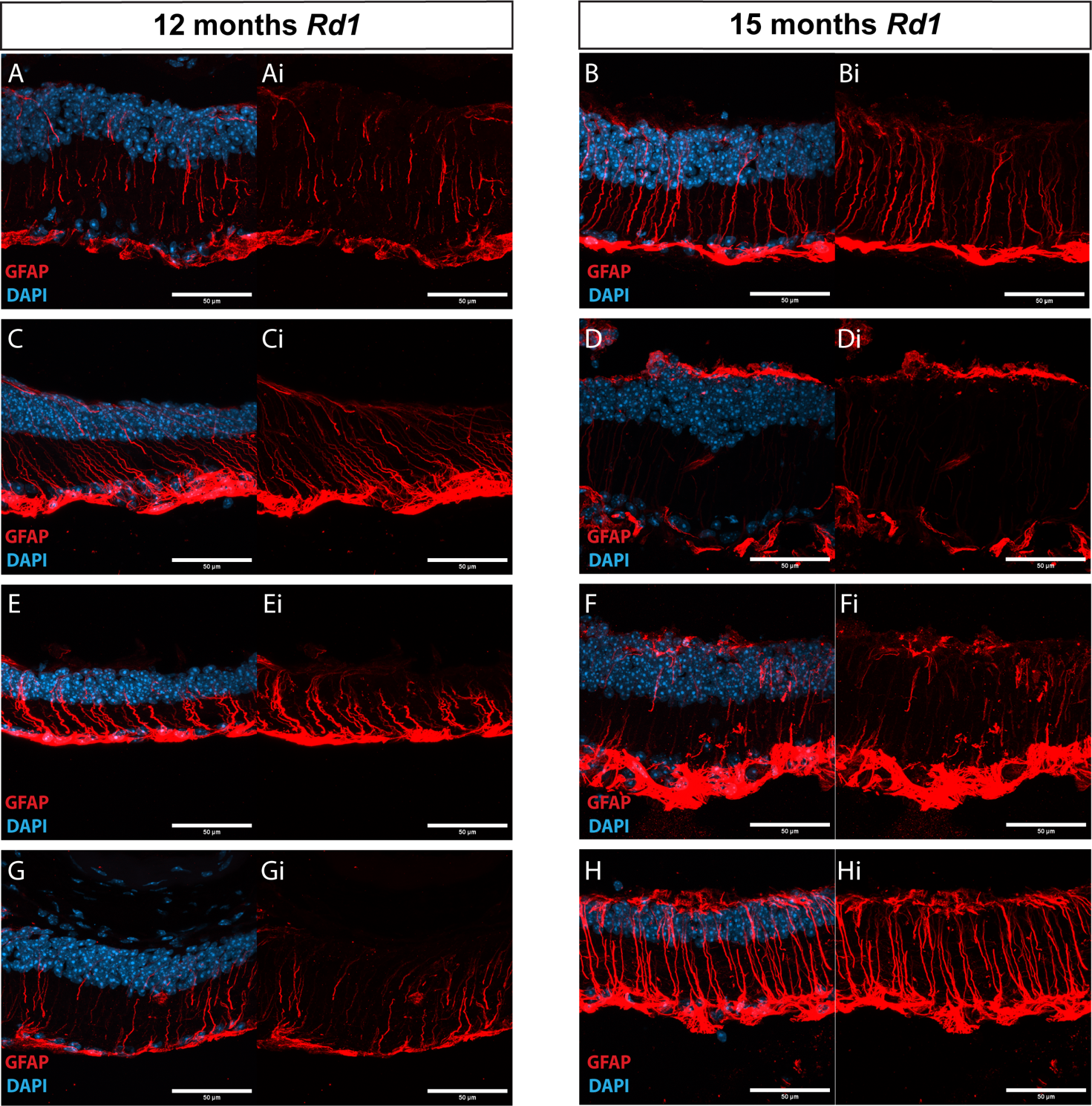


***Supplemental Figure S1: Müller glia cells show variable reactivity even in animals of same age and disease stage***

Examples from Gfap (*red*) expression in N = 4 different 12- and 15-month old *rd1* retinas (l*eft*, and *right*, respectively), showing the heterogeneity in expression and hypertrophy of Müller glial cell. Scale bar, 50 μm.


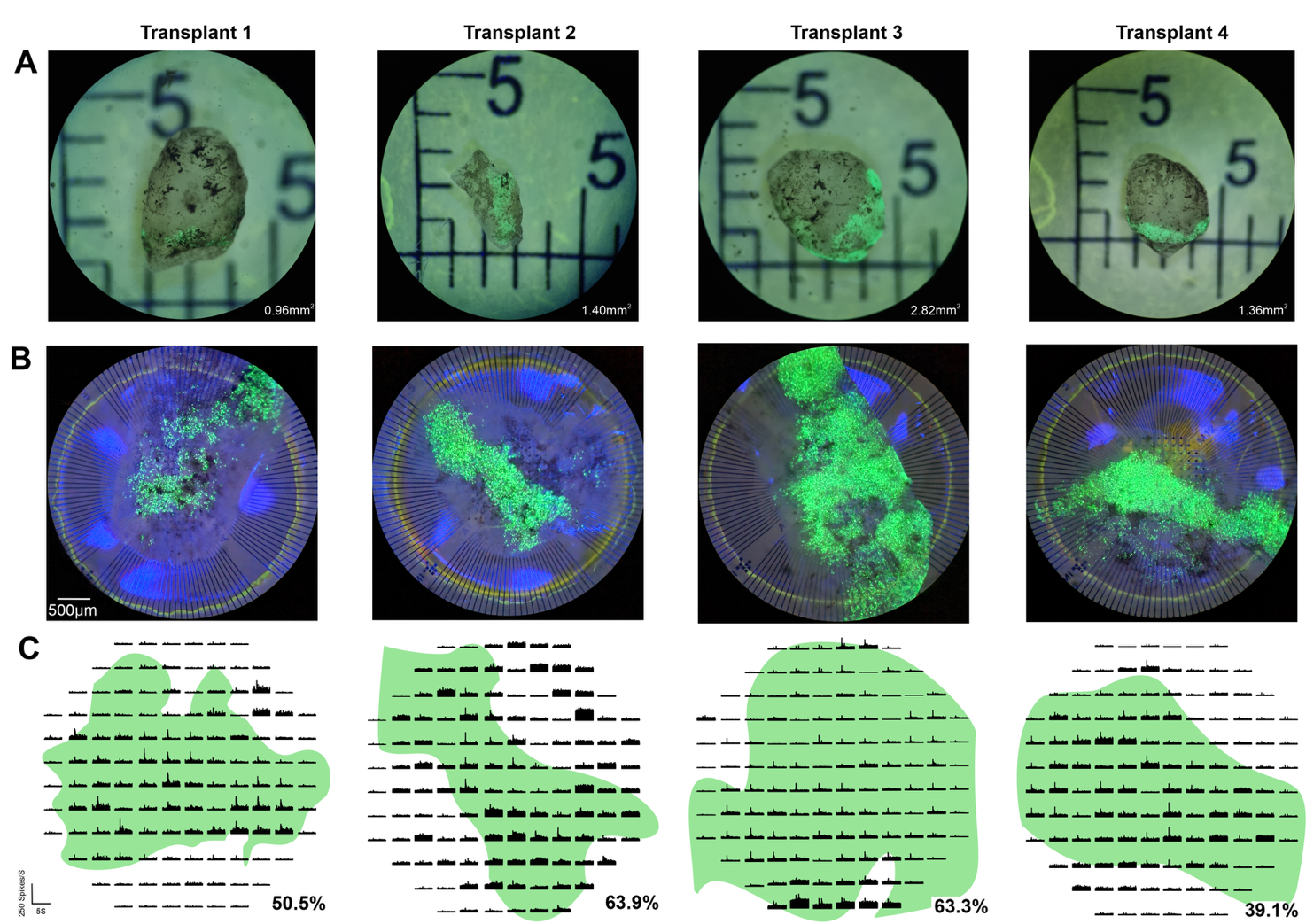


**Supplementary Figure 2 Comparison of Graft size, variability and responses in Multi Electrode Array Recordings**

**(A)** Fluorescent images of whole retinas for each of the four human cone transplants used for electrophysiological recordings. Approximate graft area (noted, *bottom right*) was determined from the intact eye cup prior to MEA processing using a graticule. Mean ± SD 1.64 ± 0.41mm^2^, N = 4 eyes. **(B)** Following dissection of the transplanted retina, regions of GFP+ hCones were centred over the electrodes and placed retinal ganglion cell layer down. Note due to variation in graft shape and size following transplantation not all electrodes can be covered by the cell mass. **(C)** PSTHs of channels in response to 10 repeats of a 1s light step from darkness, spatially reconstructed onto the arrangement of the MEA recording electrodes demonstrating that light responsive channels correlate with position of GFP+ cell mass (*green overlay*). *Bottom right*, % of single units classified as light responsive following spike sorting for each retina.


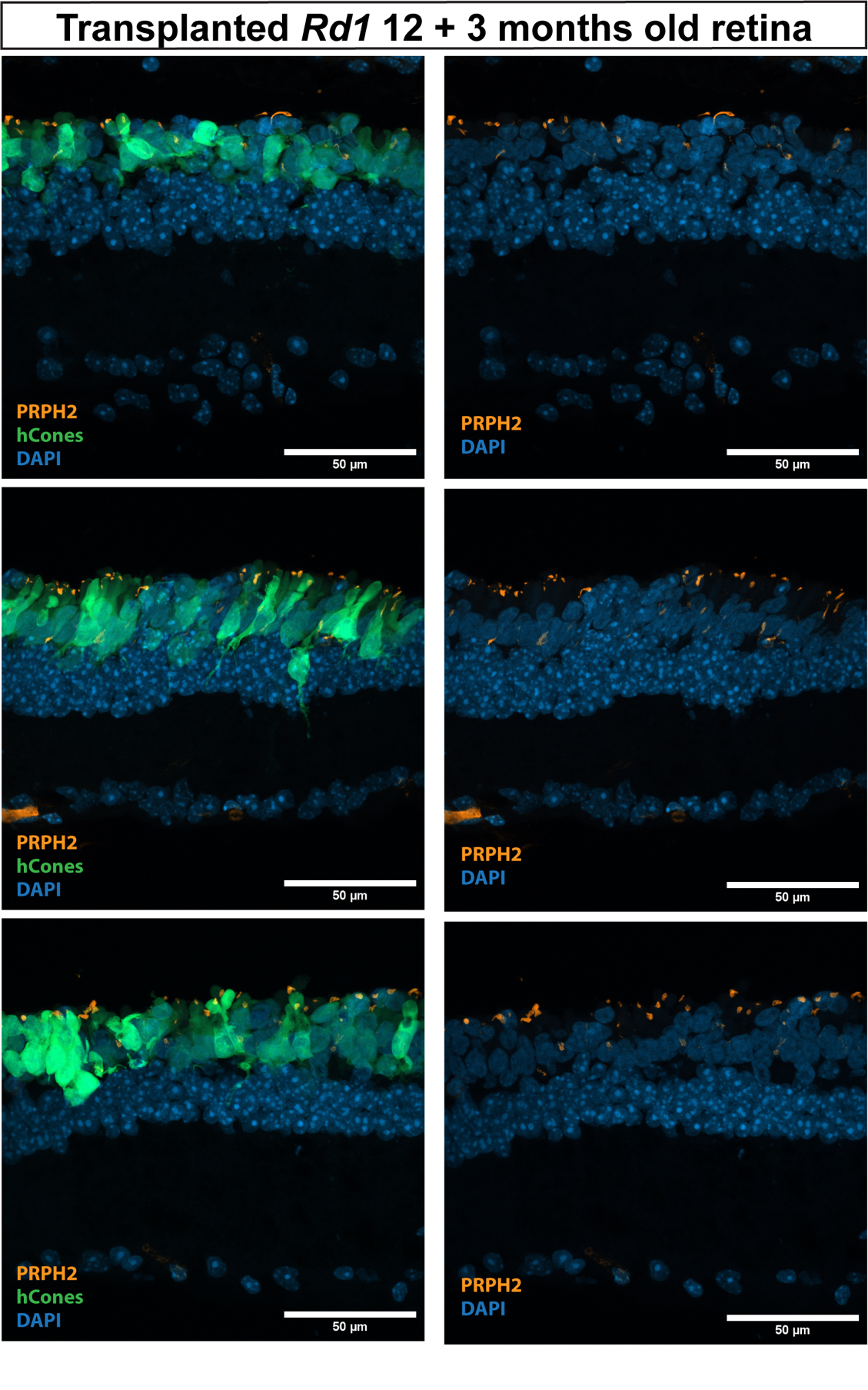


***Supplemental Figure S3: Transplanted hCones elaborate PRPH2+ segment-like structures that are typically located on the apical side of the cell mass***

**(A-C)** Retinal sections from aged *rd1* retinas transplanted with GFP+ hCones (*green*) and stained for PRPH2 (*orange*) 3 months post-transplantation. PRPH2+ buds were frequently located on the apical side of the transplanted cell mass, nearest the host RPE. Scale bar, 50 μm.

***
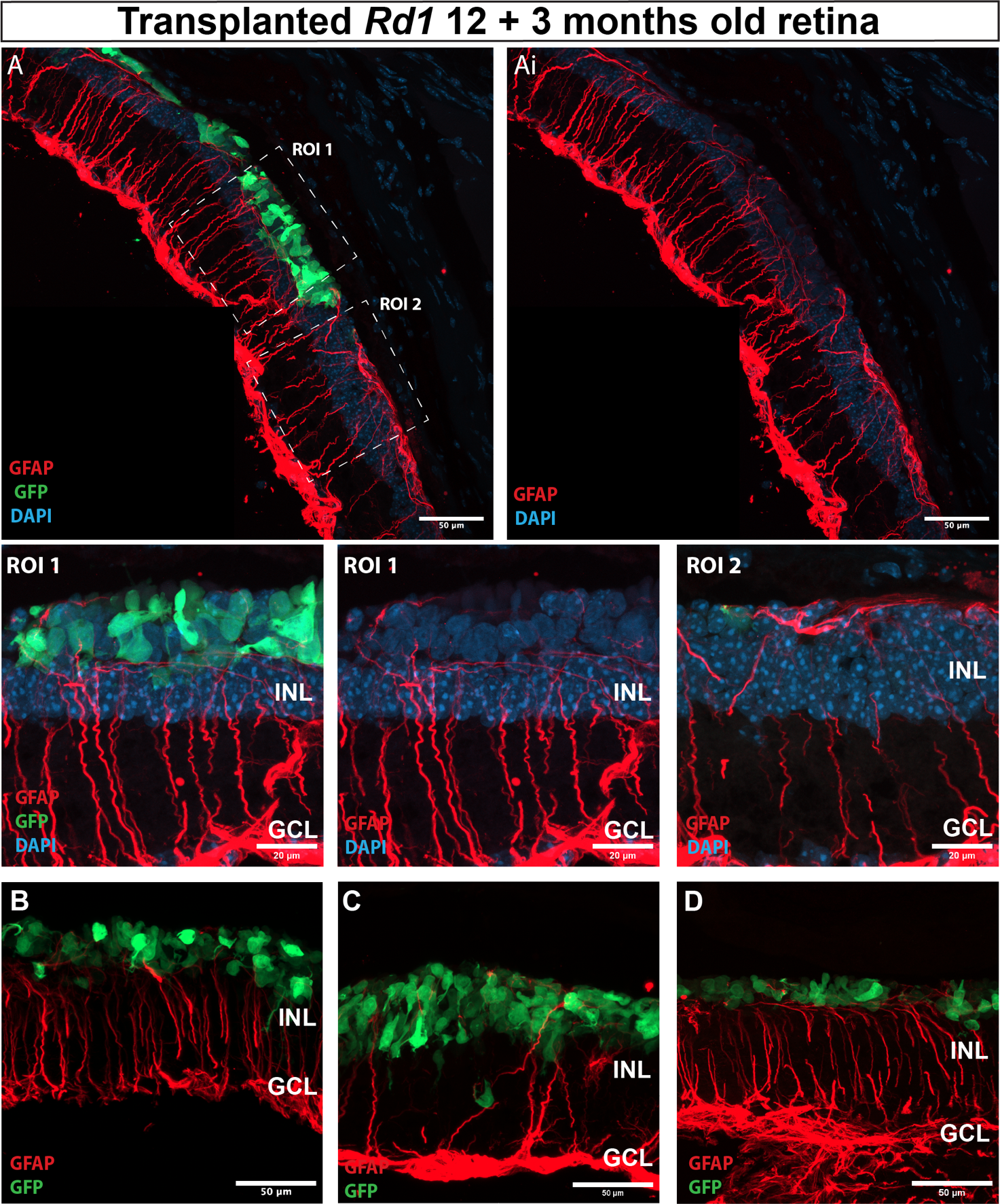
***

***Supplemental Figure S4: Müller glia cells within the recipient retina extend into and delineate the outer edge of the human cone graft***

Examples of aged *rd1* retinas transplanted with GFP+ hCones (*green*) and examined 3 months post-transplantation. Müller glia label for Gfap (*red*), indicating significant levels of reactive gliosis occurring at this stage of degeneration. (**A),** Cross section through a region of the transplanted retina, showing (*ROI 1*) host Müller glial cells (*red*) from the host retina remodelling and extending up into and through the hCone graft, frequently delineating its apical margin and incorporating the graft within the host retinal structure. Conversely, in a region adjacent to the edge of the graft (*ROI 2*), the Müller glia can be seen forming a dense matt of hypertrophic processes. (**B-D),** shows three further examples of Müller glial remodelling to encompass the transplanted hCones. Scale bar, 50 μm.

**
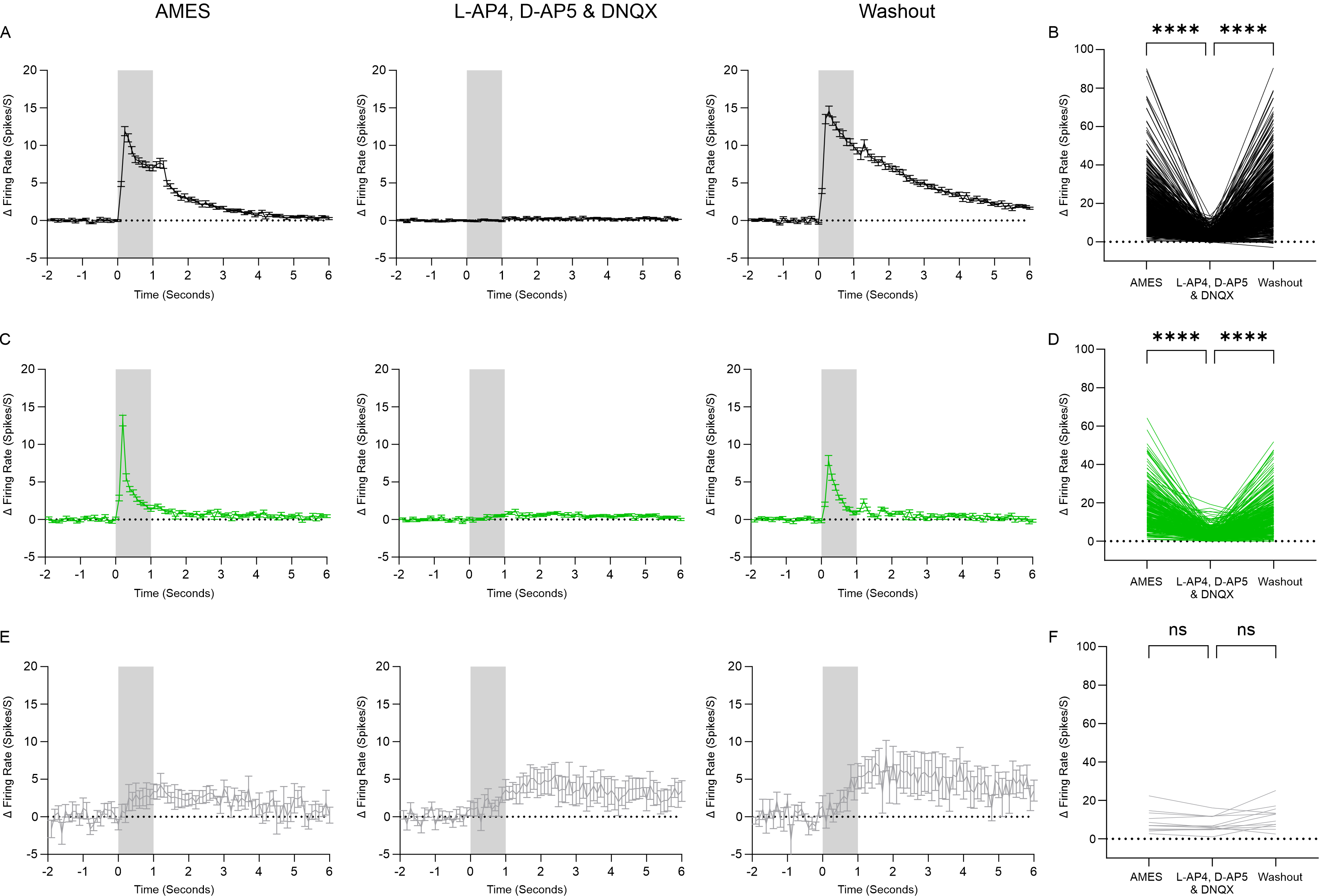
**

***Supplemental Figure S5: Single unit quantification of glutamatergic transmission of visual information at the human cone-host bipolar cell synapse***

**(A)** Average PSTH (Mean ±SEM) of all ON-type responses in aged (>1yr old) *Gnat1^-/-^* retinas (n=547 units; N = 4 retinas) demonstrates a robust light response when stimulated with a 1s light pulse from darkness (*left*), which is eradicated during application of synaptic blockers (middle) and returns following Washout (*right*). (**B**) Peak ON response amplitude of all single units in **(A)** demonstrates a significant reduction in amplitude under synaptic blockade (Mean ±SEM; 2.37 ± 0.11 Spikes/s) when compared to the AMES (18.54 ± 0.58 Spikes/s) and Washout conditions (21.52 ± 0.64 Spikes/s; 2-Way ANOVA; p < 0.001). (**C**) Average PSTH of ON-type responses in aged *rd1*+hCone transplanted retinas (n = 304 show a robust light response to the same 1s light pulse from darkness, (*left*) which is eradicated during synaptic blockade (middle) and returns upon Washout (*right)*. (**D**) Peak ON response Amplitude of all single units in C demonstrates a significant reduction in amplitude under synaptic blockade (3.42 ± 0.18 Spikes/s) when compared to the AMES (16.45 ± 0.61 Spikes/s) and Washout conditions (12.67 ± 0.58 Spikes/s; 2-Way Anova; p < 0.001). **(E)** Average PSTH of all ON-type responses in age-matched untreated *rd1* retinas (n= 12 units) demonstrates slow and sustained light responses originated from intrinsically photosensitive retinal ganglion cells when stimulated with a 1s light pulse from darkness (*left*), which is not eradicated during application of synaptic blockers (*middle*) and remains following Washout (*right*). **(F)** Peak response amplitude is not significantly different under synaptic blockade (7.68 ± 1.22 Spikes/s) when compared to AMES (8.95 ± 1.62 Spikes/s; 2 Way Anova, p = 0.10) or Washout conditions (11.29 ± 1.80 Spikes/s; 2-Way Anova; p = 0.053). **** P < 0.0001

**SUPPLEMENTARY TABLES**

| Antibodies | Source |
| --- | --- |
| Rhodospin | Sigma |
| Mouse Cone Arrestin | Merck Millipore |
| S-Ospin | Merck Millipore |
| L/M Opsin | Merck Millipore |
| Peripherin-2 (PRPH2) | Merck Millipore |
| PKC-𝛼 | Santa Cruz Biotechnology |
| Calbindin | Swant |
| Secretagogin (SCGN) | Biovendor |
| Calretinin | Abcam |
| Gfap | Calbiochem |
| Human Cone Arrestin | Novus Biologicals |
| Human Nuclei Antigen | Merck Millipore |
| CtBP2 (Ribeye) | BD Biosciences |
| mGluR6 | Custom Antibody from ThermoFisher. C-terminal synthetic peptide of mouse mGluR6 (*aa853-871*), protein sequence: KKTSTMAAPPKSENSEDAK |
| Donkey anti-mouse Alexa Fluor 488 | INVITROGEN, ThermoFisher Scientific |
| Donkey anti-rabbit Alexa Fluor 405 | INVITROGEN, ThermoFisher Scientific |
| Donkey anti-rabbit Alexa Fluor 546 | INVITROGEN, ThermoFisher Scientific |
| Donkey anti-mouse Alexa Fluor 546 | INVITROGEN, ThermoFisher Scientific |
| Donkey anti-chicken Alexa Fluor 647 | INVITROGEN, ThermoFisher Scientific |
| Donkey anti-rabbit Alexa Fluor 647 | INVITROGEN, ThermoFisher Scientific |
| Streptavidin Alexa Fluor 633 conjugate | INVITROGEN, ThermoFisher Scientific |

**Supplementary Table S1**

Supplementary Table detailing primary and secondary antibodies used.

| Animal | Untreated *rd1* mean ± SD) | *rd1*+hCones (mean ± SD) |
| --- | --- | --- |
| 1 | 0.03 (± 0.07) | 0.0 (± 0.0) |
| 2 | 0.0 (± 0.0) | 0.0 (± 0.0) |
| 3 | 0.0 (± 0.0) | 0.12 (± 0.10) |
| 4 | 0.0 (± 0.0) | 0.04 (± 0.07) |
| 5 | 0.0 (± 0.0) | 0.12 (± 0.19) |
| 6 | 0.0 (± 0.0) | 0.10 (± 0.09) |
| 7 | 0.0 (± 0.0) | 0.0 (± 0.0) |
| 8 | 0.0 (± 0.0) | 0.10 (± 0.19) |
| 9 | 0.0 (± 0.0) | 0.0 (± 0.0) |
|  | **Mann-Whitney U** | **P = 0.035** |

**Supplementary Table S2. OKR acuity measurements in untreated and hCone-treated *rd1* eyes.** Table provides the mean (± S.D.) acuity values for all untreated and hCone-treated *rd1* eyes at 3 months post-transplantation, based on 3 or 4 trials/eye.
